# Tocopherols and Tocotrienols in Common and Emerging Dietary Sources: Occurrence, Applications, and Health Benefits

**DOI:** 10.3390/ijms17101745

**Published:** 2016-10-20

**Authors:** Fereidoon Shahidi, Adriano Costa de Camargo

**Affiliations:** 1 Department of Biochemistry, Memorial University of Newfoundland, St. John’s, NL A1B 3X9, Canada; adrianoesalq@gmail.com; 2 Department of Agri-Food Industry, Food & Nutrition, “Luiz de Queiroz” College of Agriculture, University of São Paulo, Piracicaba 13418-900, Brazil

**Keywords:** tocols, edible oils, specialty oils, phenolic antioxidants, cardiovascular disease, cancer, diabetes, obesity

## Abstract

Edible oils are the major natural dietary sources of tocopherols and tocotrienols, collectively known as tocols. Plant foods with low lipid content usually have negligible quantities of tocols. However, seeds and other plant food processing by-products may serve as alternative sources of edible oils with considerable contents of tocopherols and tocotrienols. Tocopherols are among the most important lipid-soluble antioxidants in food as well as in human and animal tissues. Tocopherols are found in lipid-rich regions of cells (e.g., mitochondrial membranes), fat depots, and lipoproteins such as low-density lipoprotein cholesterol. Their health benefits may also be explained by regulation of gene expression, signal transduction, and modulation of cell functions. Potential health benefits of tocols include prevention of certain types of cancer, heart disease, and other chronic ailments. Although deficiencies of tocopherol are uncommon, a continuous intake from common and novel dietary sources of tocopherols and tocotrienols is advantageous. Thus, this contribution will focus on the relevant literature on common and emerging edible oils as a source of tocols. Potential application and health effects as well as the impact of new cultivars as sources of edible oils and their processing discards are presented. Future trends and drawbacks are also briefly covered.

## 1. Introduction

Edible oils are mainly composed of fatty acids in the form of triacylglycerols, which generate energy for the human body upon metabolism. Additionally, edible oils are sources of minor compounds such as tocopherols, tocotrienols, or both, carotenoids, and phytosterols. Tocopherols are among the most important lipid-soluble antioxidants in food as well as in the human and animal tissues. Tocopherols are found in lipid-rich regions of cells (e.g., mitochondrial membranes), fat depots, and lipoproteins such as low-density lipoprotein cholesterol. Tocopherols and tocotrienols, collectively known as tocols, are phenolic compounds. Although phenolic and polyphenolic compounds such as phenolic acids, flavonoids, anthocyanins, proanthocyanidins, and ellagitannins have received much attention due to their antioxidant activities and potential health benefits [1], tocopherols and tocotrienols and their applications need to be highlighted. Furthermore, tocols may also render positive biological effects by regulating gene expression, by signal transduction, and by modulating cell functions though modulation of protein-membrane interactions [2].

**Figure 1:**
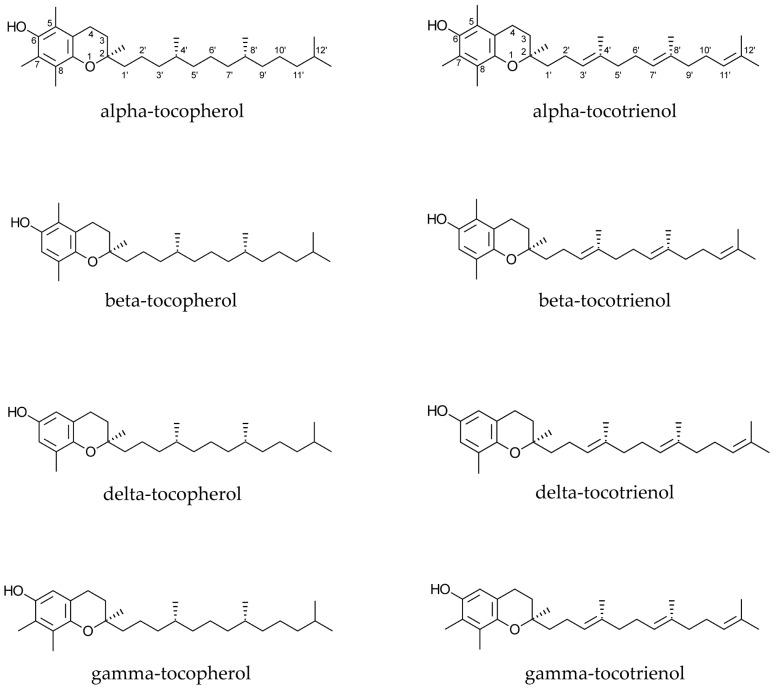
Chemical structures of tocopherols and tocotrienols.

Tocopherols and tocotrienols (Figure 1) are monophenols, and exist as four homologues (alpha, beta, delta, and gamma), which differ from each other by the number and location of methyl groups in their chemical structures.

The difference between tocopherols and tocotrienols is due to the presence of three double bonds at positions 3′, 7′, and 11′ in the side chain of the latter ones (Figure 1). Furthermore, the presence of three chiral centres is responsible for the existence of eight stereoisomers for each tocopherol, while each tocotrienol has only two stereoisomers because of the lack of chiral centres in their side chains. Discussion of structure-activity relationships has demonstrated that some isomers may be more active or sensitive than others. The same concept has been reported for different homologues.

Because of their antioxidant activity, tocols play a major role in protecting mono- and polyunsaturated fatty acids (PUFAs) from oxidation, which may also explain the high concentration of these phenolic antioxidants in highly unsaturated edible oils [3]. In addition, some edible oils may also contain tocotrienols. The lipid fraction of oilseeds and nuts is the major natural dietary source of tocopherols and tocotrienols, but their presence in fruits and vegetables is generally negligible because of their low lipid content; however, seeds and other plant food processing by-products may serve as alternative sources of edible oils with considerable contents of tocopherols and tocotrienols. The stability of tocopherols and tocotrienols may be influenced by the system in which they are present and in foods by the fatty acid composition of their lipid fraction which differs even among different cultivars of the same crop [4], by storage and processing of their feedstocks [5], and by cooking procedures employed [6].

**Figure 2:**
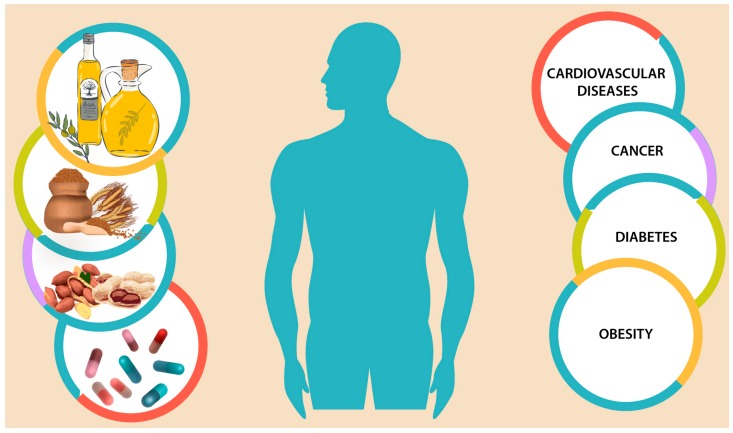
Potential health benefits through intake of tocopherols and tocotrienols.

Alpha-tocopherol, which is the most common homologue, is the major tocopherol in many edible oils as exemplified by almond [7], peanut [5], olive [8], and sunflower [9] oils. The content of gamma-tocopherol in some edible oils such as canola, corn, camelina, linseed, soybean, and walnut oils are similar or higher than that of alpha-tocopherol [10,11]. Palm oil contains three tocotrienol homologues (alpha, gamma, and delta) [12]. Deficiencies of vitamin E (the common name used for tocopherol and tocotrienol family) may include anemia, impairment of immune response, retinopathy, and neuromuscular and neurological problems; however, deficiencies are uncommon as typical diets appear to provide sufficient amounts of tocols to the body. However, as evidenced for other phenolic compounds [13,14,15,16], potential health benefits of tocopherol intake are numerous and go beyond addressing any deficiency issues (Figure 2). Therefore, to prevent certain types of cancer, heart disease, and other chronic ailments, the required intake of tocols may be much higher than their required daily intake (RDI) [17,18]. Furthermore, synthetic forms of tocopherols are not as bioavailable as their natural counterparts [19]. Therefore, discussion on common and novel dietary sources of tocopherols and tocotrienols, as well as on the identities, quantities, and stability of these biomolecules is essential in filling the gaps in the existing literature. Thus, the present article focuses on the literature (within the years of 1978 until 2016) procured from different databases (Google Scholar, Pubmed, Scielo, Scopus, and Web of Science). Common and emerging edible oils as a source of tocols are summarized. Potential application and health benefits, as well as the impact of new cultivars as sources of edible oils and their processing discards are discussed. Future trends and drawbacks are also briefly covered.

## 2. Edible Oils as Sources of Tocopherols and Tocotrienols

### 2.1. Common Edible Oils

Palm oil has overtaken soybean oil as the world’s leading vegetable oil [20]. The contents of tocopherols and tocotrienols in some common edible oils are given in Table 1. Although alpha-tocopherol is present in palm oil, gamma-tocotrienol is the major compound among other homologues. It is worth mentioning that palm oil has been reported to be the only edible oil with gamma-tocotrienol as the major lipophilic antioxidant among common ones such as castor, coconut, corn, linseed, olive, peanut, soybean, sunflower, wheat germ, and cod liver oils [21]. Soybean oil is also very important due to its high global production, especially in Brazil, China, Argentina, and USA. The content of gamma-tocopherol in soybean oil has been reported to be seven times higher than that of alpha-tocopherol, which is the most common form of this phenolic compound [21]. Similar to soybean oil, alpha-tocopherol is not the most prominent tocol in canola or rapessed oil, in which the content of gamma-tocopherol was up to 2.7-fold higher than alpha-tocopherol [11]. Therefore, it is worth paying attention to the content of gamma-tocopherol in both soybean and rapeseed oils and to their potential technological and health effects. Alpha-tocopherol comprises more than 93% of the tocols in sunflower oil. Furthermore, none of the previous oils had alpha-tocopherol as the main tocol; therefore, the assumption that alpha-tocopherol is the most common form should be made with caution because, at least in terms of vegetable oil production, this information may not be representative. Among the three most consumed edible oils, gamma-tocotrienol is the most prominent tocol in the first (palm oil), while gamma-tocopherol is the most prominent one in the second (soybean oil) and third (canola oil). However, alpha-tocopherol is the main tocopherols only in the fourth most consumed oil (sunflower oil), but oils from sunflower cultivars obtained by mutagenesis and genetic recombination [22] had 77% and 68% of beta- and delta-tocopherols, respectively.

The Mediterranean diet has been in the spotlight due to its association with a lower incidence of cancer, cardiovascular ailment, and Parkinson’s and Alzheimer’s diseases [32]. Olive oil is the characteristic lipid source of the Mediterranean diet. Additionally, chefs from the finest restaurants all over the world love to emphasize the use of olive oil in their preparations. The most notable difference between extra virgin and traditional olive oils are due to differences in their manufacturing processes. The industrial process of extra virgin olive oil (EVOO) includes pressing of the olive fruit in a solvent-free process; moreover, no heat is applied, which is beneficial for retention of its bioactive phenolic compounds, including tocols. Like certain wines, some EVOOs display label information to confirm that they belong to a “Protected Designation of Origin” and therefore follow very strict requirements defined by specific legislations. EVOO has alpha-tocopherol as its major tocol homologue. A recent study [8] demonstrated that the content of alpha-tocopherol varies greatly in commercial olive oils. The same study suggested that considering a daily intake of 30 g, olive oil could supply an average of 50% of the required daily intake (RDI) for alpha-tocopherol (12 mg of vitamin E). A food product can be considered a source of vitamin E as long as its consumption offers a supplement of at least 15% of the vitamin E RDI; therefore, the commercial extra virgin olive oil producers are allowed to use this health claim.

As mentioned before, four tocopherols and their corresponding tocotrienols are known. However, not all studies provide the contents for individual tocols, which may lead to an incorrect interpretation of the data. Usually, identification and quantification of tocopherols have been carried out using HPLC. This technique requires authentic standards for identification and quantification. The number of tocopherol homologues is very low when compared with other phenolic antioxidants such as phenolic acids and flavonoids (including hydrolysable tannins, proanthocyanidins, and anthocyanins), which makes their identification very simple. However, because of co-elution, there are several studies in which gamma- and beta-tocopherols have been reported together (gamma- plus beta-tocopherol). In the last few years, the number of studies employing hyphenated techniques such as HPLC-MS^n^ has been growing; however, it is still difficult to find data for several feedstocks. The contents of specific tocopherol homologues may differ among cultivars and crop years [4] for the same feedstock. Peanuts are among the most popular oilseeds in the world [33], and peanut oil contains alpha-, beta-, delta-, and gamma-tocopherols [4]. The study of Shin et al. [4] provided the content of the four homologues in the lipid fraction of 151 samples of US grown Runner peanuts from two crop years provided by ten cultivars (normal-, mid-, and high-oleic). According to their data, the total tocopherol content was not different among normal, mid-, and high-oleic cultivars, but the content of alpha-tocopherol did vary among Runner cultivars classified by their oleic acid content. Differences were also found among different cultivars for all tocopherol homologues, and production year influenced the content of alpha- and beta-tocopherols. The contents of alpha- and gamma-tocopherols reported by these authors was similar for these two homologues. In contrast, Runner peanuts from Brazil [5] had higher contents of gamma-tocopherol, which was followed by alpha-, delta-, and beta-tocopherols, therefore suggesting the role of climatic and stress conditions as well as the soil quality on the distribution of tocopherol homologues.

### 2.2. Specialty and Underutilized Edible Oils

The content of alpha- and gamma-tocopherols in grapes, guava, melon, passion fruit, pumpkin, and tomato seed oils (Table 2) was reported by da Silva and Jorge [34]. Although beta- and delta-tocopherols were quantified together, gamma-tocopherol was nonetheless the most abundant homologue in guava, melon, pumpkin, and tomato seed oils. Furthermore, alpha-tocopherol was not detected in the oils extracted from passion fruit and tomato seeds. In another study [35] reporting the contents of all tocopherol homologues, delta-tocopherol was most abundant in passion fruit seed oil. The tocopherol content was also reported for seed oils of four varieties of pumpkin. Beta-tocopherol was present in only two samples and made the lowest contribution to the total content. However, alpha- and delta-tocopherols, which were reported along with beta-tocopherol in some studies, rendered the second highest content in one sample, which was higher than that for alpha-tocopherol. Lipids and carbohydrates (including fibres) accounted for 29.0% and 30.5% of fresh weight, respectively. Thus, papaya seeds are a viable feedstock for oil extraction [36]. The content of unsaturated fatty acids in papaya seed oil was 77.5%. Papaya (*Carica papaya* L.) consumption and processing generates large amounts of seeds as by-products. Besides being a good source of carotenoids, alpha-tocopherol was the major tocol homologue in the lipid fraction of papaya seed oil, followed by delta-, gamma-, and beta-tocopherols. The seeds from citrus fruits also contain a large amounts of lipids. Among them, orange, tangerine, and lemon seeds contained 41.5%, 41.6%, and 34.9% [37] oil and alpha-tocopherol made the highest contribution among the remaining homologues. Blueberry, blackberry, raspberry, and grapeseed oils are examples of specialty oils. Because of their pleasant flavour, they have been used mostly in the cosmetic industry as an ingredient in different formulations. The quantity of polyphenols in these oils may be negligible when comparted with those of their starting material (e.g., seeds). However, tocol homologues may be present in specialty oils as natural dietary sources of liposoluble antioxidants [38,39]. These feedstocks are well recognized as sources of phytochemicals in both the water soluble and lipid soluble forms; thus, interest in different starting materials including native fruits has been growing.

Jubuticaba (*Myrciaria jaboticaba* (Vell.) O. Berg) and juçara fruits (*Euterpe edulis* Mart.) are species from the Brazilian Atlantic Forest; the first one being grown mainly by local farmers and the fruits are produced at most twice per year [44]. Jabuticaba is consumed as such because of seasonality, while some parts of the fruit are used to make products such as juices, jams, and alcoholic beverages, which generates different by-products like seeds and skins. Because of seasonality and shelf-life issues [45], juçara has been used to produce pulp, therefore generating agro-industrial by-products. Jabuticaba, juçara, and their by-products were reported to have tocopherols [44]. According to Morales et al. [46], alpha-tocopherol was the major homologue in the lipid fraction of non-fermented and fermented jabuticaba. Although beta-, gamma-, and delta-tocopherols were identified and quantified, their data demonstrate that the concentration of alpha-tocopherol was up to twelve times higher than that found for delta-tocopherol and 3.2-fold higher than the remaining homologues (beta- plus gamma- plus delta-tocopherol). Inada et al. [44] also reported alpha-tocopherol as the most prominent tocopherol in jabuticaba among beta- and gamma-tocopherols. According to these authors, alpha-tocopherol also made the highest tocol contribution in juçara. Because juçara contains a high lipid content [45], it may be considered as a novel source of specialty oil. Additionally, Darnet et al. [47] reported that delta-tocopherol was not detected in açaí (*Euterpe oleracea*) and alpha-tocopherol accounted for 97.2%–97.7% of the total tocopherols in samples collected from four different locations.

Tamarind (*Tamarindus indica* L.) originated in Africa, but it is primarily grown in India. In Brazil, the highest production and consumption takes place in the north and northeast of the country. Tamarind seed oil has alpha-, beta-, gamma-, and delta-tocopherols, although gamma-tocopherol has been found to contribute 68.5% of its total tocopherol content [43]. *Syagrus oleracea*, *Syagrus romanzoffiana*, and *Acrocomia aculeata* (commonly known as guariroba, jerivá, and macaúba) were studied by Coimbra and Jorge [48]. Regardless of the starting material, the content of alpha-tocopherol was higher in the oil extracted from the pulp than in the kernel, which may be explained by the higher content of unsaturated fatty acids in the former. In another study [40], delta-tocopherol was demonstrated to be the second most prominent tocopherol in the pulp and seeds of guariroba and jerivá, but not in macaúba, in which gamma-tocopherol made the second highest contribution in the pulp but was not detected in the seed.

Jatoba (*Hymenaea courbaril* L.) is native to Brazil and is typically found in Cerrado; the second largest biome in South America. The fruit is a source of fibre, showing contents of 50.0% and 72.1% in the pulp and seeds, respectively. The highest lipid content is found in the seed (5.8%) as compared to the pulp (1.9%). The content of alpha-tocopherol, the most abundant homologue in jatoba pulp and seeds was higher in the oil extracted from the seed as compared to the pulp, but the difference was not as significant as that found for gamma-tocopherol, which was also present in higher concentrations in the oils extracted from the seed. The remaining homologues, beta- and delta-tocopherols, were not detected in any of the tested samples [41]. *Annona crassiflora* Mart (also known as pinha or fruta do conde) is also typical of the Brazilian Cerrado. According to Luzia and Jorge [42], the lipid yield of *Annona crassiflora* Mart seeds was 28.8%. The same study also demonstrated that all tocopherol homologues were present in the oil extracted from the seeds, although the content of delta-tocopherol seemed to be negligible. According to these authors, gamma-tocopherol made the highest contribution.

## 3. Impact of New Cultivars on the Content of Tocopherols and Tocotrienols

Specialty, novel, and underutilized edible oils are highlighted here as potential sources of tocopherols and tocotrienols; however, regardless of the starting material (novel vs. common edible oil), the interest in genetic breeding to produce novel cultivars with a higher content of a particular biomolecule has been increasing. The great variability in the content of tocopherols, among different cultivars, has been reported for peanuts, for example [4]. It seems clear that the food industry has been focusing on the development of edible oils with a higher oleic-to-linoleic ratio because this increases their stability of the oil against oxidative processes, and this has been discussed for different feedstocks, including palm [49], soybean [50], canola [51], sunflower [51], and peanuts [52,53,54], among others. However, due to their critical effect on health, the influence of genetic modification on the content of minor components such as tocopherols and tocotrienols should also be considered.

Abidi and co-workers [55] reported that canola modification changed the content of tocopherols, and the greater concentration change was noted in alpha- and gamma-tocopherols as compared to delta-tocopherol. It has been accepted that the relative concentrations of tocols in different feedstocks may be used to distinguish them according to their botanical origin [1]. The content of alpha-tocopherol has been found to contribute the most in sunflower oil [11,25], peanuts [21,26], and olive oil [11,21]. The presence of tocotrienols in tocotrienol-free vegetable oils has been proposed as a method to investigate their authenticity [56]; however, the increasing amount of new varieties may affect this evaluation method. In general, alpha-tocopherol is also dominant in safflower oil, but a recent study reported the development of a new variety, which accumulates a higher content of gamma-tocopherol, and this demonstrates the problems associated with using the dominant tocopherol as an evidence of oil adulteration. In addition, while some studies have reported the absence of tocotrienols in olive, sunflower, and soybean oil [56]; Schwartz and co-workers [11] detected their presence in olive and sunflower oils. Additionally, the presence and enhancement of tocols preferentially in the form of tocotrienols using genetic approaches was discussed by Clemente and Cahoon [57]. Gamma- and delta-tocotrienols, which were not detected in wild-type soybean seeds, were present in transgenic soybean seed cultivars. The same study also showed that alpha-tocotrienol, which was not present in non-germinated transgenic soybean seeds, was detected in some transgenic lines after three days of germination [58]. It is clear that most studies have used HPLC itself to investigate the presence of tocopherols and tocotrienols; however, in the last few years, some research teams [39,59] have used HPLC-MS for identification and quantification purposes, which is useful in providing a complete picture for the presence of these high-valued molecules. The identification and quantification of all eight tocols in every starting material as well as the clarification of their presence or absence is useful for providing a database, which would support geneticists in the development of new cultivars.

## 4. Current and Potential Applications of Tocopherols and Tocotrienols

Tocopherols and tocotrienols are procured from deodorizer distillate in the refining of vegetable oils [60,61,62]. Mixed tocopherols from soybean oil processing are generally used for stabilizing oxidatively-sensitive lipid supplements such as those from marine oils and other products. In addition, tocopherol-rich oils, such as germ oils from oat [63], barley [64] and wheat [65], may be mixed with other oils to stabilize them [61]. Among the tocotrienol-rich oils, palm oil [28], annatto [66], and rice bran oil [67] are important, and these are often used in product enrichment. Considerable amounts of tocols are removed during the refining process of edible oils. The loss of tocopherols during the deodorization step has been estimated to exceed one-third of the original tocopherol concentration [68]. A recent study [69] has shown that residual minor compounds, including tocopherols and tocotrienols, influence the oxidative stability of commercially refined vegetable oils. Therefore, it is certain that even small concentrations of these antioxidants influence the stability of the final product.

Huang et al. [70] evaluated the antioxidant activity of alpha-tocopherol in bulk corn oil stripped of its natural tocopherols and reported that alpha-tocopherol had the maximum capacity in inhibiting hydroperoxide formation at 100 ppm. Additionally, alpha-tocopherol inhibited hexanal formation in a concentration-dependent manner. In another study [71], alpha- and delta-tocopherol and alpha-, delta-, and gamma-tocotrienols (100–5000 ppm) were investigated for their antioxidant capacity in stripped corn oil. Lower concentrations of alpha-tocopherol and alpha-tocotrienol were more efficient than higher concentrations in preventing the formation of hydroperoxides, and a prooxidant effect was found in concentrations higher than 700 ppm. In contrast, delta-tocopherol, delta-tocotrienol, and gamma-tocotrienol did not show any prooxidant effects and exhibited antioxidant activity in a concentration-dependent manner. Wagner and co-workers [72] reported that the antioxidant activity of alpha-, gamma-, and delta-tocopherols and alpha-, beta-, gamma-, and delta-tocotrienols in coconut fat was concentration-dependent at both 60 and 160 °C. Additionally, these authors showed that gamma-tocotrienol was most effective in preventing lipid oxidation. Other studies have also highlighted the superior antioxidant capacity of gamma-tocotrienol [71,73] compared with other tocols. The order of antioxidant activity of tocopherols and tocotrienols is dependent on several factors such as their concentration in the system, oxidation temperature, and interactions with other molecules, which may render synergistic or antagonistic effects. However, according to the literature [38], in fats and oils, gamma-tocotrienol is generally more effective than alpha-tocotrienol, while tocotrienols generally exhibit higher protection towards oxidation than that of their corresponding tocopherols. The present contribution detected a lack of data on tocotrienols in novel dietary sources of tocols; therefore, further research is necessary to fill this apparent gap in the existing literature. Tocopherols may act as antioxidants in a synergistic manner with other polyphenols [74] and phospholipids [75]. Khan and Shahidi [75] evaluated the antioxidant effectiveness of individual phospholipids. Phosphatidylcholine (PC) had higher antioxidant capacity than that of phosphotidylethanolamine (PE) in borage triacylglycerols (TAG), while the reverse was observed for evening primrose TAG. The same study also demonstrated the synergism between alpha-tocopherol and phospholipids in borage and evening primrose TAG. According to these authors, the most effective combination in borage TAG was that of PC and alpha-tocopherol, while PE with alpha-tocopherol exerted the best effects in evening primrose TAG, therefore suggesting that alpha-tocopherol may be used in combination with different phospholipids depending on the TAG contents.

Regarding potential application in food emulsions, gamma-tocopherol, ethylenediaminetetraacetic acid (EDTA), and ascorbyl palmitate were examined for their antioxidative protection in fish oil-enriched salad dressing [76]. Gamma-tocopherols exerted a partial reduction in the formation of lipid hydroperoxides. Ascorbyl palmitate showed a prooxidant effect at higher concentrations; however, these effects were not observed when a combination of all three antioxidants was used. The combination of antioxidants almost completely inhibited the oxidation over six weeks of storage at room temperature. Zou and Akoh [66] evaluated the ability of annatto tocotrienol-rich fraction (TRF) and palm TRF for their antioxidant capacity in menhaden oil and structured lipid-based infant formula emulsions. Annatto TRF contained only tocotrienols (gamma- and delta-tocotrienols), whereas palm TRF had tocopherols and tocotrienols (alpha-tocopherol and alpha-, beta-, delta-, and gamma-tocotrienols). These authors demonstrated that annatto TRF was more efficient than palm TRF in inhibiting the oxidation of both tested materials. Furthermore, the combination of alpha-tocopherol with annatto was also tested to evaluate if its presence would attenuate the antioxidant capacity of annatto TRF, but no effect was observed. In another study [77], the same authors tested the antioxidant capacity of selected antioxidants (namely alpha-tocopherol, beta-carotene, ascorbyl palmitate, ascorbic acid, citric acid, and their combinations) in structured lipid-based infant formulas (IF) and found a synergistic effect between alpha-tocopherol and beta-carotene. Zhang et al. [78] demonstrated that tocols from the lipophilic fraction of black rice bran were effective in inhibiting the oxidation of fish oil emulsion and showed higher efficacy than that found with anthocyanins. Parenteral nutrition (intravenous feeding) is used to provide energy to patients with different diseases, including impairment of the gastrointestinal tract. Essential fatty acids are among the nutrients delivered using parenteral nutrition. To understand the potential biological effects of tocols, Xu et al. [79] evaluated the identities and quantities of tocopherols and tocotrienols in commercial parenteral lipid emulsions containing medium-chain triacylglycerols, soybean, olive, and fish oils, as well as their mixtures. The contents of tocopherols varied among different samples, whereas the contribution of tocotrienols was minimal or non-existent. The same research team [80] reported that the level of tocopherols in different tissues remained at the baseline or only slightly increased after vein infusions of these emulsions using an animal model. Furthermore, the deposition of tocopherols varied among different tissues (e.g., liver, heart, lung, kidney, and adipose tissue), with lung and liver exhibiting the highest tocopherol deposition.

Fish, red meat, and poultry are among the most important animal sources of protein in the human diet. Additionally, these feedstocks and their commercial products are also rich sources of lipids, which are susceptible to oxidation [81]. Horn and co-workers [82] evaluated the addition of gamma-tocopherol to fish oil-enriched energy bars and found that prooxidant and antioxidant properties were dependent on the concentration of tocopherols added to each product. Gamma-irradiation reduces the microbial contamination of fish, red meat, and poultry, but it also induces lipid oxidation. According to Kanatt et al. [83], the addition of alpha-tocopherol prior to irradiation (2.5 kGy) lowered the oxidation level of chicken meat. Active packaging with antioxidant compounds has also been studied as a means of preventing lipid oxidation. Barbosa-Pereira and co-workers [84] tested five commercial additives that contained tocopherols to prevent the oxidation of salmon. The additive containing the highest concentration of tocopherols (90.2%) was the most effective in extending the shelf-life of salmon. Additionally, incorporation of antioxidants in plastic matrices (low-density polyethylene) reduced lipid oxidation of salmon muscles by up to 40%, therefore suggesting that active films could be applied to extend the shelf-life of high-lipid foods. Chen et al. [85] evaluated the release of tocopherols and quercetin in different packaging films and demonstrated that tocopherols presented a faster release compared to quercetin. Active packaging has been thoroughly studied; however, technological issues, such as structural changes in the packaging materials, are still in need of further research [86].

*N*-nitrosamines (NAs), which are formed by the reaction of primary and secondary amines and nitrosating agents [87], are carcinogenic to animals and possibly humans; thus, nitrates, nitrites, proteins, peptides, and amino acids may also participate in the reaction [88]. *N*-nitrosamine formation in cured meat has been found to be dependent on the degree of unsaturation of the adipose tissue of the feedstock [89], and this may be related to a higher oxidation susceptibility and formation of malondialdehyde and related oxidation products. According to Kurechi and co-workers [90], malondialdehyde greatly influences the formation of NAs and suggested a possible carcinogen-promoting effect of malondialdehyde. In addition, protein oxidation has been found to affect the formation of NAs upon heating [88], which was attenuated by alpha-tocopherol addition. Other studies [91,92,93] with bacon and cured sausage have reported that alpha-tocopherol was efficient in decreasing the concentration of NAs.

One of the most important parameters for consumers in deciding to buy a food product is its appearance. Customers judge the quality of foodstuffs on various characteristics such as size, texture, smell, and colour. The red colour of meats, such as beef and pork, signifies their freshness; however, during the storage time, oxymyoglobin is oxidized to metmyoglobin [94] and a brownish colour starts to appear in place of the desired bright red color. Djenane and co-workers [95] reported that alpha-tocopherol improved the inhibition capacity of vitamin C against the formation of metmyoglobin of beef steaks packaged in modified atmosphere during storage. Furthermore, a recent study [74] demonstrated that alpha-tocopherol may have a synergistic effect in combination with phenolic extracts from açaí and grape by-products in protecting methyl linoleate against metmyoglobin-initiated oxidation.

Cod liver oil is used as a supplement and for medical purposes due to the presence of health-promoting polyunsaturated fatty acids (PUFA), such as eicosapentaenoic acid (EPA) and docosahexaenoic acids (DHA), and vitamin A. However, cod liver oil is prone to oxidation because of its high PUFA content. The liver of Atlantic cod fed mixed tocopherols (alpha-, delta-, and gamma-tocopherols) showed an improved oxidative status [96]; therefore, these liposoluble antioxidants play a critical role in the stability and sensory quality of foods and lipid supplements.

Tocopherols and tocotrienols also contribute to the prevention of several chronic ailments; therefore, fortifying different food products with tocols may be beneficial to consumers. However, the lipophilicity of tocopherols may be a challenge in hydrophilic systems such as non-alcoholic beverages. Consequently, nanoemulsions have been suggested as a technological approach to overcome this problem [97]. Because tocopherols may be employed to improve the stability of food systems and to prevent the onset of chronic diseases, many different challenges remain about their final application.

## 5. Stability and Bioavailability of Tocopherol and Tocotrienols

The identities, quantities, and some potential health benefits of tocopherols and tocotrienols are highlighted in different sections of this contribution. In this, we focus on the scavenging ability of tocols towards reactive oxygen species (ROS) and the prevention of atherosclerosis, cancer, and generation of endogenous *trans* fatty acids. The potential management and the prevention of diabetes and obesity are also discussed. Additionally, tocols potentially attenuate the risk of neurological and inflammatory diseases [98,99]. In fact, Etminan and co-workers [100] conducted a meta-analyses by analyzing studies published between 1966 and 2005 and suggested that alpha-tocopherol may attenuate the risk of Parkinson’s disease. Another meta-analysis correlated the intake of tocopherols with a preventive effect towards Alzheimer’s disease [101]. The benefits mentioned thus far appear to be dependent on the identities and intake quantities of tocols; therefore, a discussion about their stability is relevant. As mentioned before, the stability of tocopherols and tocotrienols in food systems may be influenced by the fatty acid composition of the lipid fraction, by storage and industrial processing of their feedstocks [5], and by the cooking procedures employed [6].

Frying is among the most used cooking procedures, both by the food industry and in home cooking. The degree of unsaturation has been shown to influence the oxidative stability of fats and oils but may also affect the stability of tocopherols. According to Corsini et al. [27], the highest loss of total tocopherols was noted in sunflower oil (52%) as compared with palm oil (8%) after deep frying of frozen cassava chips, which was attributed to the lowest degree of unsaturation of the latter. Additionally, alpha-tocopherol was the most sensitive and delta-tocopherol the least sensitive in sunflower oil. Likewise, alpha-tocopherol decreased the most in palm oil, followed by beta-tocopherol. In agreement with the above study, de Camargo et al. [5] showed that gamma irradiation induced lipid oxidation in peanuts, and alpha-tocopherol was the most sensitive homologue extracted from the lipid fraction. In another study [102], alpha-tocopherol was also found to be the most sensitive to the blanching process conducted by the peanut industry. The above-mentioned studies [5,27,102] identified and quantified all four forms of tocopherol in their starting material, which is useful for comparative purposes. Tree nuts are also important sources of tocopherols [103]; gamma-irradiation decreased the content of alpha-tocopherol in walnuts by up to 87.4% when subjected to a dose of 8 kGy, and its concentration was under the detection limit in samples submitted to 10 kGy [104]. Gamma-irradiation is used to improve the microbiological safety of different food products [105], but it has detrimental effects on sensory attributes of high-lipid foods [45,105], as well as in fat soluble compounds such as tocopherols. Additionally, heating and light exposure also have a similar deleterious effect [106]. Therefore, regardless of the process or condition, exposure of sources of tocopherols and tocotrienols to detrimental situations should be minimized. Furthermore, the synergism of alpha-tocopherol and polyphenols is well documented [107,108], and the presence of polyphenols in the lipid system has been shown to be helpful in the retention of natural tocopherols [109]; thus, the combination of tocopherols and polyphenols may be an alternative for the industry.

Bioavailability plays a crucial role in the biological activity of natural products. Phenolic compounds such as phenolic acids, flavonoids, and proanthocyanidins exist both in the soluble form, comprising molecules in the free, esterified, and etherified as well as in the insoluble-bound form, the latter by being linked to cell wall materials [13,16,110]. Soluble proanthocyanidins are readily available, while insoluble-bound proanthocyanidins must be catabolized in the colon. Additionally, it has been suggested that proanthocyanidins with higher degrees of polymerization have lower bioavailability [111]. The molecular weight among different tocols does not vary much when compared to the variation found for different proanthocyanidins; however, the literature documents different bioavailability among different tocopherol and tocotrienol homologues.

According to Saito et al. [112], the higher efficacy of alpha-tocopherol as compared with that found for alpha-tocotrienol against glutamate toxicity in vivo is explained by the higher bioavailability of the former. The uptake of alpha-tocopherol is mediated by alpha-tocopherol transfer protein and gene mutations may diminish plasma alpha-tocopherol concentration [113], thereby increasing the need for tocopherol supplementation. Hosomi and co-workers [114] demonstrated the affinity of different tocols with alpha-tocopherol transfer protein and concluded that this is a determinant for their plasma level and, in turn, their biological activity. Moreover, alpha-tocopherol bioavailability has been shown to be lower in individuals with metabolic syndrome as compared with healthy adults, apparently because of greater inflammation and oxidative stress, which may limit small intestinal alpha-tocopherol absorption, impair hepatic alpha-tocopherol trafficking, or both. Therefore, a higher alpha-tocopherol requirement for individuals with metabolic syndrome has been suggested [115]. More recently, Drotleff et al. [116] investigated the human oral bioavailability and pharmacokinetics of tocotrienols from tocotrienol-rich barley oil and palm oil formulations. These authors concluded that the absorption of total tocotrienols from the former was higher, possibly due to its greater content of alpha-tocotrienol.

More than 8000 compounds from different classes, including phenolic compounds such as phenolic acids, flavonoids, proanthocyanidins, hydrolysable tannins, and flavonoids are currently known. Among the phenolic compounds, tocols comprise eight known homologues; however, some studies do not clarify as to whether all of the compounds were detected or investigated. Because the biological activity is not only dependent on the presence of tocopherols and tocotrienols but also on their quantities and identities, further studies should focus on the identification of all possible tocol forms to anticipate potential health benefits and technological application.

## 6. Tocopherols and Tocotrienols in Health Promotion

### 6.1. Tocopherols and Tocotrienols in the Prevention of Cardiovascular Diseases

Potential health benefits of tocols include prevention of certain types of cancer, heart disease, and other chronic ailments (Table 3). Phenolic antioxidants act as chain breakers through inhibition of lipid peroxidation. High levels of low-density lipoprotein cholesterol LDL-c are a risk factor for the onset of CVDs, as the presence of oxidized LDL-c is also involved as an early event in the pathogenesis of atherosclerosis, a condition where plaque inside the arteries may impair the blood flow and increase the risk of coronary heart disease. Although phenolic acids and flavonoids, including proathocyanidins and anthocyanins, inhibit the oxidation of LDL-c [15,16,117], the ability of different phenolic compounds in inhibiting the oxidation of LDL-c is related to their lipophilicity. This has been demonstrated by the lowest inhibition of lipid peroxyl radical species of gallic acid as compared with epigallocatechin gallate [118]. In fact, lipophilised phenolic compounds have shown improved antiradical activity against ROS, and this has been achieved through esterification with different fatty acids [119]. However, all forms of tocopherols and tocotrienols have a long lipophilic tail [120] that bind them to membrane cells, which is particularly important in the plasma as well as in the mitochondrial membranes. In addition, alpha-tocopherol, which has an affinity with the apolar medium, acts in a synergistic manner with phenolics that present a higher affinity with the polar fraction in liposome model systems [108]. Liposomes have been used as a valuable tool to mimic cell membranes [107]; therefore, results may be extended to biological model systems. The synergistic effect of alpha-tocopherol with other phenolics has been explained by the probable regeneration of the first one in a complex interaction with phenolics present in the polar fraction [108].

The major lipophilic antioxidant present in plasma and LDL-c is alpha-tocopherol [121]. A recent study attributed the slow in vitro oxidation rate of human LDL-cholesterol as being dependent on the depletion of lipid-soluble antioxidants such as tocopherol, therefore suggesting its critical role in the resistance of LDL-c to oxidation [15]. Furthermore, the role of alpha-tocopherol in decreasing the susceptibility of LDL-c to oxidation has also been demonstrated in human trials [122], which may be related to its ability to decrease systemic oxidant stress in vivo in humans [123]. Furthermore, a recent meta-analysis study concluded that tocopherol reduces myocardial infarction in interventional trials [124]. According to the World Health Organization [125], more than 30% of deaths worldwide are attributed to CVDs.

Consumption of *trans* fatty acids increases the risk of coronary heart disease; hence, their elimination in food is necessary [151]. The hydrogenation process has been used by the industry to extend shelf-life by converting the polyunsaturates to less unsaturated counterparts. These fats are sometimes present in certain fast foods, margarines, bakery products, and crackers [151,152]. Enzymatic interesterification has been employed to produce zero-*trans* shortenings and margarines [153]; however, the formation of *trans* fatty acids may still occur during the deodorization of vegetable oils [154]. Dietary *trans* fatty acids are correlated with increases of LDL-c in healthy adults. To minimize oxidation of LDL-c, consumption of natural rich sources of tocopherols and tocotrienols is considered helpful.

It is well established that the antioxidant activity of different tocols depends on their chemical structures; however, the literature has been focused mainly on the action of alpha-tocopherol. Although this homologue is most common, the antioxidant activity of the remaining homologues should not be ignored. Peroxynitrite, which is generated by the reaction of nitric oxide and superoxide anion, has been implicated in the vascular endothelial function [155]. According to McCarty [156], peroxynitrite may be scavenged by gamma-tocopherol. Thus, it is essential to pay attention in the preventive action of beta-, delta-, and gamma-tocopherols, as well as their corresponding tocotrienols.

### 6.2. Tocopherols and Tocotrienols as Adjuvants in Cancer Treatment and Prevention

According to the International Agency for Research on Cancer (IARC) [157], cancer is among the leading diseases worldwide. The same report showed that lung, female breast, colorectal, and stomach cancers account for more than 40% of all cases diagnosed worldwide. Tobacco was highlighted as the most important risk factor for cancer development, but other factors such as specific infections, obesity, excessive sunlight exposure, as well as certain occupational exposures have also been mentioned. Additionally, mycotoxins produced by different fungi are also among possible carcinogens. Mycotoxins may be found in different food products [158,159,160], including oilseeds, cereals, and milk; however, avoiding consumption of these products may or may not be a realistic option. DNA damage induced by different mycotoxins has been demonstrated in vitro and in vivo [127,161]. However, alpha-tocopherol was found to exhibit a protective effect towards zearalenone-induced DNA fragmentation using three different cell lines [127]. Therefore, the benefits of different sources of tocopherol may overcome potential risks posed by the presence of mycotoxins.

Surgery, chemotherapy, radiation, or any combination of the three, are the most common treatments for cancer. The role of tocols in preventing certain types of cancer has been supported by both in vitro and in vivo studies [136,162]. Campbell and co-workers [128] reported the cell growth inhibitory capacity of gamma-tocopherol using different colon cell lines. The percentage of cell death and apoptosis differed among cell lines, which was attributed to the different molecular characteristics of the cells. The recognition of the potential anti-cancer activity of alpha-tocopherol using different cell lines, as well as its safety towards normal cells, has led to the interest in the development of targeting delivery systems, which were found to have therapeutic efficacy, as evidenced both in vitro and in vivo [129]. A recent report [130] suggested that even oxidized tocols may be able to reduce tumor cell viability in vitro. Cancer prevention has been associated with a healthy diet and many individuals are becoming vegetarian because they associate meat consumption, especially cured meat products, as a health concern because of the possible increase of the risk of colon cancer development [163]. Pierre et al. [131] conducted a biological trial and reported that alpha-tocopherol reduced colon carcinogenesis biomarkers associated with colon carcinogenesis that were chemically induced by cured meat consumption. Furthermore, epidemiological studies have associated the supplementation of alpha-tocopherol with a reduced chance of gastric cancer development [132]. In fact, the effect of alpha-tocopherol on prostate cancer and mortality has been confirmed in an 18-year post-intervention follow-up study [133], thus suggesting a long-term positive effect of alpha-tocopherol consumption.

Lung and breast cancers are the most prevalent forms of diseases [157] and the former is listed as the most common type of cancer in men while the latter is most prevalent in women. Menkes et al. [134] evaluated the correlation of serum tocopherol with the risk of lung cancer and reported that individuals who developed lung cancer in the almost one-decade study had lower serum tocopherol concentration than those who did not have cancer. Additionally, individuals with serum levels of tocopherol in the lowest quintile had a 2.5 times higher risk of lung cancer than those with levels in the highest quintile. Different tocol homologues, namely delta-tocopherol as well as alpha-, beta-, and gamma-tocotrienols were effective apoptotic inducers for human breast cancer using cell lines [135], but the same was not found for alpha-, beta-, and gamma-tocopherols. According to Wada et al. [136], delta-tocotrienol showed a greater antiproliferative effect in human hepatocellular carcinoma HepG2 cells, which was followed by beta-tocotrienol, while both alpha- and gamma-tocotrienols showed the lowest, but equivalent antiproliferative effect. However, another study suggested that alpha-tocopherol may be beneficial for the treatment of breast cancer due to the alteration of alpha-tocopherol-associated protein (TAP) expression observed in human breast epithelial cells during breast cancer development [137]. A recent meta-analysis suggested that severe alpha-tocopherol deficiency could increase breast cancer risk [138]. Because of controversial results in this matter, the role of tocopherol in cancer treatment and prevention requires further attention and research.

Chemotherapy has long been used in cancer treatment, but side effects such as nephrotoxicity, neurotoxicity, hepatotoxicity, cardiotoxicity, as well as gastrointestinal and pulmonary toxicity have been reported [164]. Additionally, it has been hypothesized that drug-induced oxidative stress may explain the hepatotoxicity and cardiotoxicity [139,165]. Recent reports have discussed the role of food phenolics in preventing oxidative damage by scavenging different ROS [15,111,166]. Anthracycline (ANT), which is used to treat many cancers such as leukemias, breast, and lung cancer, undergoes redox cycling in which hydrogen peroxide, hydroxyl, and superoxide radicals are generated due to the presence of ANT-Fe^2+^ complexes. Because tocopherol has been found to decrease doxorubicin-induced hepatotoxicity in rats [139], presumably because of its antioxidant capacity, as well as its ability in scavenging different ROS, consumption of different sources of tocopherol during chemotherapy may help preventing ROS-induced side effects. Additionally, gamma- and delta-tocotrienols rendered a synergistic effect against MCF-7 human breast cancer cells when combined with tamoxifen [140]. The authors suggested that these tocols may be used to diminish the required dose of tamoxifen, which is used in the treatment of breast cancer, therefore potentially decreasing its side effects. However, adequate human studies are still needed to support their findings.

Radiotherapy, or exposure to radiation, may be used alone or in combination with surgery and chemotherapy to treat cancer. Several reports have used the DNA strand breakage method as a biomarker for investigation of ROS-induced oxidation in vitro [14,15,16,166]. Irradiation induces DNA damage in cancer cells and impairs their growth and multiplication presumably through oxidation, which generates ROS. Although normal cells may also be affected by the treatment, they have a greater ability in repairing themselves and overcome exposure to radiation. Exposure to gamma-ray and X-ray irradiation may induce single- and double-strand breaks. Furthermore, DNA damage may cause chromosomal aberrations, which have been associated with cancer development. Even low doses of gamma-irradiation may impair the bone marrow function, which has been related to the development of leukemia. According to Mayer et al. [167], the ability of human lymphocytes in vitro in rejoining from X-ray-induced DNA double-strand break depended on the age, with older subjects presenting a lesser ability in overcoming DNA damage than the younger ones. Another study [168] demonstrated that a small population of cells was not able to repair from X-ray induced DNA damage, and these cells were more common in older individuals. Furthermore, DNA damage taking place in human white blood cells in vitro appeared to be related to consumption of dietary antioxidants [169]. Additionally, in a recent study [141], human umbilical vein endothelial cells were subjected to gamma-irradiation where gamma-tocotrienol was found to attenuate DNA double-strand breaks. Moreover, the same work showed an increase in the expression of the DNA repair RAD50, therefore highlighting the importance of gamma-tocotrienol and its sources as a potential therapeutic strategy to reduce irradiation-induced genetic diseases. Because exposure to irradiation for cancer treatment is not an option for the patient, consumption of different sources of tocopherol may be helpful in mitigating deleterious effects in normal cells by increasing their ability in repairing from irradiation-induced DNA damage.

### 6.3. Diabetes

Diabetes is involved as part of the complications and cause of metabolic syndrome in which the body cannot produce insulin or make a proper use of it, thereby impairing the control of the body’s blood sugar level. Type 1 diabetes is developed when the immune system attacks the beta cells of the pancreas, killing the cells. This attack jeopardizes insulin production, which increases the blood sugar level. Moreover, type 2 diabetes is not characterized by the lack of insulin but in the inability of the body to use it properly; therefore, type 2 diabetes is also called insulin insensitivity. Depending on the severity of the disease, physical activity and a dietary plan may be successful in the management or prevention of type 2 diabetes, but insulin and medications may also be required. In some countries such as Brazil, anti-hyperglycemics are provided by the government to the population free of charge; therefore, diabetes management may also become an economic burden. Furthermore, side effects due to continuous treatment are an additional inconvenience. In light of this, plant food phenolics have attracted much interest due to their potential role in the prevention and management of type 2 diabetes [111,166,170], and efforts in discovering and isolating natural compounds have been in the spotlight [171]. Diabetes may lead to neuropathy as well as skin and eye complications; therefore, patients with diabetes are more likely to develop cataract. A higher level of serum tocopherol has been associated with reduced age-related cataract risk [172].

Several phenolic compounds may bind alpha-amylase and alpha-glucosidase, which prevent glucose absorption [173]; however, the enzyme inhibition ability has been found to vary by compound. The literature screening did not show tocols as potential inhibitors of these enzymes. Thus, the anti-diabetic effect of tocopherols may stem from other biochemical mechanisms. Peroxisome proliferator-activated receptors alpha, gamma, and delta (PPARα, PPARγ, and PPARδ) are ligand-regulated transcription factors that play essential roles in energy metabolism, and their ligand compounds have been used to treat diabetes [142]. Fang et al. [142] demonstrated that alpha- and gamma-tocotrienols activated PPARα, while delta-tocotrienol activated PPARα, PPARγ, and PPARδ. Furthermore, the tocotrienol-rich fraction of palm oil improved body glucose utilization and insulin sensitivity of diabetic mice, which was explained by the selective regulation of PPAR target genes. A recent study [143] with poloxamer-407 (PX-407)-induced type 2 diabetic Wistar rats, at which hyperglycemia response was induced due to loss of β-cell sensitivity to glucose, suggested the tocopherol-mediated antihyperglycemic effect of *Cucurbita* pepo seed oil. Observational studies have suggested the protective effect of alpha-tocopherol against the development of diabetes in humans subjects [144], although no additional benefits have been found with tocopherol supplementation [174]. This information suggests that dietary sources of tocopherols, but not their supplementation, along with other strategies such as weight loss, dietary intervention, and physical activity may be useful for the prevention and management of diabetes. Because experimental and clinical evidence demonstrated a higher generation of ROS in both types of diabetes [175], the role of oxidative stress in the onset of complications caused by diabetes has also been a concern. The antioxidant potential of tocopherols and tocotrienols against ROS-induced damage is well established; however, in this contribution, we focused on relevant literature and the potential prevention mechanisms of diabetes through the consumption of dietary sources of tocols.

### 6.4. Obesity

The World Health Organization (WHO) defines obesity as an abnormal or excessive fat accumulation in a level that presents a risk to health [176]. Obesity increases the risk of developing CVDs, cancer, and diabetes. The estimated annual increase rates in the prevalence of overweight preadolescence and adolescent children in the United States, Brazil, and China were 0.6%, 0.5%, and 0.2%, respectively [177]. Excess weight, along with the increase in the prevalence of diabetes and hypertension, has been highlighted among the non-communicable diseases (NCDs) responsible for 72% of all deaths in Brazil in 2007 [178]. The above-mentioned increases were associated with unfavorable diet changes; therefore, encouraging consumption of healthy foods could help reduce the number of NCDs. A study [179] was conducted over an eight year period with 148,579 subjects from developing countries. The authors concluded that, in contrast to what was expected, the number of overweight women exceeded the number of underweight women in most countries. Additionally, Monteiro et al. [180] reported that, regardless of the region (less or more developed), education was inversely associated with obesity in women. This study suggests that education may be decisive for correct and balanced diet planning, which may decrease the chance of becoming obese and suffering from its related diseases.

The role of tocopherols in the development of CVDs, cancers, and diabetes has been discussed in this contribution. As already mentioned, these health issues are related to the prevalence of obesity. Additionally, non-alcoholic fatty liver disease (NAFLD) has been associated with the increase in the prevalence of obesity, diabetes, and metabolic syndrome [181]. Liver function and histologic changes have been significantly improved by consuming alpha-tocopherol in patients with NAFLD [145]. According to Botella-Carretero et al. [146], the serum concentration of alpha-tocopherol in morbidly obese patients was inversely associated with body mass index. Additionally, alpha-tocopherol levels were also significantly lower in obese children than in non-obese subjects [147]. Although other phenolic compounds [111,166,170] have been reported to act as inhibitors of lipase with a potential prevention role in obesity, there seems to be a lack of any literature reports of this nature with tocopherols.

Fats, oils, and lipid-rich foods have a negative connotation among those who may wish to lose weight or stay in shape. However, lipids are not just a source of condensed energy, but are also important carriers of essential biomolecules that may help in health promotion and disease risk reduction. In fact, a study on rats [148] demonstrated that gamma-tocotrienol (60 mg/kg of body weight/day) reduced body fat mass induced by different doses of glucocorticoid. Uto-Kondo et al. [149] evaluated the effect of tocotrienol-rich fraction from palm oil against adipocyte differentiation in 3T3-L1 cells. These authors suggested that alpha- and gamma-tocotrienols suppressed the differentiation of preadipocytes into adipocytes, potentially preventing obesity. However, strong epidemiological evidence addressing the relationships between the intake of tocotrienols, and body weight reduction in humans are still necessary [182].

Red blood cells (RBCs) move along the blood flow in the circulatory system and collect oxygen in the lung and deliver it to the body tissues. Physical activity in combination with a proper diet is highly recommended to maintain or lose weight. During exercise, the RBC population increases due to the higher oxygen demand; however, their contact with oxygen may also accelerate oxidative damage. In a human trial conducted during acute exhaustive exercise in collegiate women [183], it was demonstrated that RBC had lower alpha-tocopherol concentration after exercise. The same study demonstrated that alpha-tocopherol supplementation resulted in a steady alpha-tocopherol level in RBCs. Because obesity has been related to a lower level of tocopherol, overweight and obese individuals must pay additional attention to dietary sources of tocopherols, especially when exercising in order to inhibit oxidative damage to RBCs.

### 6.5. The Role of Tocopherols and Tocotrienols against Endogenous Formation of Trans Fatty Acids

A recent review [152] brought to discussion the constant concern on the adverse effects on human health caused by the consumption of *trans* fatty acids (TFA), which may include an increased chance of developing cardiovascular disease, diabetes, and cancer. Furthermore, pro-inflammatory responses, due to the consumption of large amounts hydrogenated oils, which are sources of TFA, were also reported. Although the natural presence of TFA has been attributed only to some bacteria or ruminants, this report highlighted the possible endogenous formation of TFA in humans. According to the authors, free radical stress appears to be responsible for both fatty acid peroxidation and their possible *cis-trans* isomerization. Additionally, thiyl and nitrogen dioxide radicals were highlighted as the most biologically relevant species catalyzing the *cis-trans* fatty acid isomerization. Therefore, their scavenging by tocopherol is deemed beneficial.

Smoking has been linked to an increased risk of developing CVDs and cancer. Furthermore, air pollution, which contains nitrogen oxides, has been recognized as a possible cause of cancer. Additionally, nitrogen dioxide as a free radical is regarded as a possible cause for a higher concentration of *trans* arachidonic acid in cells, in tissues, and in the systemic circulation of smokers as compared with non-smokers, thus suggesting a nitrogen dioxide-catalyzed isomerization in vivo [152,184]. Unhealthy habits such as smoking may be avoided, which may not be possible due to contact with pollution. Likewise, avoiding the aging process is obviously not an option. Zambonin et al. [185] reported the occurrence of TFA in rats fed a diet free of TFA, demonstrating fatty acid endogenous isomerization. In a follow up study [186], a significant increase in unnatural TFA content of the erythrocyte, the kidney, and the heart was observed. Their data demonstrated a significantly higher content of TFA in the hearts and kidneys of old rats as compared with the younger rats, suggesting that TFA generation is also related to the aging process. The lack of isomerization in the liver, which was not dependent on the age of the rats, was attributed to the high tocopherol content in their livers.

Glutathione peroxidase (GSH), a tripeptide formed by glutamic acid, cysteine, and glycine, is an important antioxidant enzyme for biological systems. During its redox reaction an intermediate thiyl radical is formed through hydrogen atom donation from the thiol group of cysteine, which is the active site of the molecule. Thiyl radicals can induce *cis-trans* isomerization in monounsaturated fatty acids (MUFA) [187] and polyunsaturated fatty acids (PUFA) [188] and appears to be independent of the position and the length of the chain [150]. One of the most important functions of the lipoprotein membranes of the cells brings about their selective permeability, which manages what can cross the membrane and participate in biochemical reactions within the cell. Membrane lipoproteins contain unsaturated fatty acids, which are susceptible to radical-induced reactions. Konings and co-workers [189] reported the X-ray irradiation-induced damage in liposome model systems. According to these authors, alpha-tocopherol was able to decrease detrimental effects, and the protective effect was higher than that found for reduced glutathione (GSH) and cysteamine (MEA). Chatgilialoglu et al. [150] demonstrated that thiyl radicals induce *cis-trans* isomerization in phospholipid bilayers, which changes their permeability. The same study also demonstrated that alpha-tocopherol was able to inhibit the isomerization process. Therefore, the effects of tocopherol in TFA generation in vivo warrants additional research.

## 7. Conclusions

Edible oils are rich sources of tocols; however, much research is still needed to provide the identities and quantities of individual homologues, especially in terms of the presence and contents of tocotrienols in specialty oils. This may be achieved by applying modern methods, including mass spectrometry. The current applications in food systems have been focused on the antioxidant capacity of tocols as applied individually or in combination with other antioxidants, which may render synergistic effects. Tocols are also related to several health benefits and play a crucial role in the prevention of certain types of cancer and cardiovascular diseases. Additionally, tocopherols and tocotrienols participate in important biochemical mechanisms related to the onset of diabetes and obesity. However, despite the fact that there is no evidence for any potential harmful effects of tocols, caution should still be exercised if recommending supplementation, especially for the dosage considered. Because of the generally greater antioxidant activity of tocotrienols as compared with their tocopherol homologues, the development of high-tocotrienol cultivars by agronomic geneticists should be pursued.

## Figures and Tables

**Table 1:** The contents of tocopherols and tocotrienols (mg/100 g of oil) in some common edible oils.

Oil [Reference]	Alpha-T	Beta-T	Gamma-T	Delta-T	Alpha-T3	Beta-T3	Gamma-T3	Delta-T3
Barley [23,24]	14.2–20.1	0.60–1.90	3.50–15.1	0.90–4.60	46.5–76.1	nd–12.4	8.50–18.6	0.50–2.6
Coconut [11,21]	0.20–1.82	tr–0.25	tr–0.12	nd–0.39	1.09–3.00	nd–0.17	0.33–0.64	nd–0.10
Corn [11,21]	18.0–25.7	0.95–1.10	44.0–75.2	2.20–3.25	0.94–1.50	nd	1.30–2.00	nd–0.26
Cottonseed [25,26]	30.5–57.3	0.04–0.30	10.5–31.7	tr	nr	nr	nr	nr
Linseed [11,21]	0.54–1.20	nd–tr	52.0–57.3	0.75–0.95	nd	nd	nd	nd
Olive [11,21]	11.9–17.0	nd–0.27	0.89–1.34	nd–tr	nd–tr	nd	nd	nd–tr
Palm [21,27,28]	6.05–42.0	nd–0.42	tr–0.02	nd–0.02	5.70–26.0	nr–0.82	11.3–36.0	3.33–8.00
Peanut [21,26]	8.86–30.4	nd–0.38	3.50–19.2	0.85–3.10	nd	nd	nd	nd
Rapeseed [11,21]	18.9–24.0	nd–tr	37–51	0.98–1.90	nd	nd	nd	nd
Rice bran [25,29]	0.73–15.9	0.19–2.5	0.26–8.00	0.03–2.70	0.84–13.8	tr–2.6	1.74–23.1	0.14–2.53
Safflower [21,26]	36.7–47.7	nd–1.20	tr–2.56	tr–0.65	nd	nd	nd	nd
Sesame [11,30]	0.24–36.0	0.28–0.80	16.0–57.0	0.17–13.0	tr	nd	0.34	nr
Soybean [21,31]	9.53–12.0	1.00–1.31	61.0–69.9	23.9–26.0	nd	nd	nd	nd
Sunflower [11,25]	32.7–59.0	tr–2.40	1.40–4.50	0.27–0.50	0.11	nd	tr	tr
Wheat germ [11,21]	151–192	31.2–65.0	tr–52.3	nd–0.55	2.5–3.6	nd–8.2	nd–1.85	nd–0.24

References are given in brackets by number appearing at the end of the manuscript; nd: not detected; nr: not reported; and tr: trace.

**Table 2:** The content of tocopherols (mg/100 g) in specialty seed oils.

Oil [Reference]	Alpha-T	Beta-T	Gamma-T	Delta-T
Camelina [11]	3.80	0.09	72.0	1.50
Grape [34]	11.8	nr	60.1	nr
Guariroba [40]	1.19	nd	nd	0.78
Guava [34]	45.8	nr	93.1	nr
Jatoba [41]	88.6	nr	16.9	nr
Jerivá [40]	1.10	0.10	nd	0.78
Lemon [37]	102	2.20	1.33	19.0
Macaúba [40]	1.44	0.08	nd	0.79
Melon [34]	20.5	nr	250	nr
Orange [37]	300	nd	nd	18.6
Papaya [36]	5.18	0.21	0.18	1.89
Passion fruit [35]	nd	5.40	16.7	27.9
Pinha [42]	1.20	0.33	12.3	0.02
Pumpkin [34]	7.30	nr	294.5	nr
Soursop [34]	22.1	nr	7.10	nr
Tamarind [43]	1.24	0.70	3.82	0.02
Tangerine [37]	116	2.22	nd	19.2
Tomato [34]	nd	nr	32.9	nr

References are indicated in brackets and appear at the end of the manuscript; nd: not detected; nr: not reported.

**Table 3:** Potential health benefits reported though experiments in vitro and in vivo.

Test Material	Main Outcomes	Disease/Function	Reference
Alpha-T	Synergism of alpha-tocopherol with hydrophilic phenolic extracts from *Centella asiatica*, açaí, and grape by-products in liposome model systems was dependent on the mixture ratio	Potential preventive oxidation in lipid membranes	[74,108]
Alpha-T	Healthy men who received at least 400 IU/day showed lower levels of oxidized LDL-cholesterol	Cardiovascular diseases	[122]
Alpha-T	Supplementation with alpha-tocopherol alone (400–800 IU/daily), but not in combination with other antioxidants, decreases fatal myocardial infarction by about 20% according to a meta-analysis evaluating randomized clinical trials	Cardiovascular diseases	[124]
Gamma-T	Generation of stable carbon-centered adducts through the nucleophilic 5-position thus trapping membrane-soluble electrophilic nitrogen oxides as evaluated in liposome model system and human LDL-cholesterol in vitro	Cardiovascular diseases	[126]
Alpha-T	Reduction of zearalenone-induced DNA fragmentation and apoptotic body formation using Vero, Caco-2 and DOK cell lines	Cancer	[127]
Alpha- and gamma-T	Gamma-T, but not alpha-T, reduced malignant colon cancer cell proliferation (SW480, HT-29, HCT-116, and HCT-15 cells) and the effectivity was dependent on the molecular characteristics of the cells	Cancer	[128]
Alpha-T	Polymeric micelles (targeting ligand-modified TOC-DOX) containing alpha-T (TOC) and doxorubicin (DOX) reduced proliferating cell nuclear antigen expression in tumor tissues of HER2/neu-positive SK-BR-3 tumor-bearing mice	Cancer	[129]
Alpha- and gamma-T3	Both oxidized and nonoxidized gamma-T3 decreased the viability of MCF‑7 breast cancer cells in vitro and the former was more effective	Cancer	[130]
Alpha-T	Alpha-T reduced the number of mucin-depleted foci (a precancer lesion) in rats fed a diet containing cure meat	Cancer	[131]
Alpha-T	Alpha-T (10 mg/day) decreases the risk of gastric cancer development by 24% as evaluated by a meta-analysis	Cancer	[132]
Alpha-T	Alpha-T (50 mg/day) prevented the onset of prostate cancer and its related mortality showed long-term decrease (18-year post-trial)	Cancer	[133]
Alpha-T	Lower levels of alpha-T were associated with lung cancer development in humans	Cancer	[134]
Delta-T, alpha-, beta- and gamma-T3	The mentioned tocols, but not alpha-, beta-, and gamma-T, were apoptotic inducers in both estrogen-responsive MCF7 and estrogen-nonresponsive MDA-MB-435 human breast cancer cell lines in vitro	Cancer	[135]
Alpha-, beta-, delta-, and gamma-T3	All tocotrienols showed anticarcinogenic effects in lung and liver cancer in mice	Cancer	[136]
Alpha-, beta-, delta-, and gamma-T3	Delta-T3 showed higher antiproliferative effect in human hepatocellular carcinoma HepG2 cells in vitro but increased CYP1A1 gene expression raising a concern of potential carcinogenic effect in some cases	Cancer	[136]
Alpha-T	Alpha-T-associated protein (TAP) expression was associated with the reduction of alpha-T levels in human breast tumour samples	Cancer	[137]
Alpha-T	Severe deficiency of alpha-T was associated with higher risk of breast cancer development as evaluated through a meta-analysis	Cancer	[138]
Alpha-T	Alpha-T (200 IU/kg/week) reduced the doxorubicin-induced hepatotoxicity in rats and decreased malondialdehyde levels in their liver	Cancer	[139]
Gamma- and delta-T3	Both gamma- and delta-T3 showed synergistic effects against MCF-7 human breast cancer cells when combined with tamoxifen in proportion 1:1	Cancer	[140]
Tocotrienol-rich fraction (TRF) from palm oil, alpha-T, alpha-, gamma-, and delta-T3	All of them combined with tamoxifen (1:1) showed synergistic effects towards MDA-MB-435 cells proliferation	Cancer	[140]
Gamma-T3	Gamma-T3 inhibited DNA double-strand breaks in gamma-irradiated human umbilical vein endothelial cells, positively influenced the expression of the DNA-repair gene RAD50 and diminished radiation-induced RAD50 suppression	Cancer	[141]
Alpha-, delta- and gamma-T3	Peroxisome proliferator-activated receptors (PPAR) were modulated by alpha-T3 (PPARα), gamma-T3 (PPARα), and delta-T3 (PPARα, PPARγ, and PPARδ)	Diabetes	[142]
Tocotrienol-rich fraction (TRF) of palm oil	PPAR target genes of diabetic mice were regulated by TRF of palm oil, improved their glucose levels and sensitivity to insulin	Diabetes	[142]
Alpha-, beta-, delta-, and gamma-T	Antidiabetic properties tocopoherols are related to their docking to dipeptidyl peptidase IV and peroxisome proliferator-activated receptor gamma and the interaction nature (hydrogen bond, hydrophobic, or Pi-Pi) is dependent on the interacting residue	Diabetes	[143]
Alpha-T	Insulin-dependent diabetes mellitus (IDDM) with lower levels of alpha-T showed higher prevalence of IDDM after 4–14 years baseline examination	Diabetes	[144]
Alpha-T	Liver function and histologic changes in patients with nonalcoholic fatty liver disease, including nonalcoholic steatohepatitis were improved by alpha-T as evaluated by a meta-analysis of randomized controlled trials	Obesity	[145]
Alpha-T	Body mass index of morbidly obese patients was inversely correlated with their alpha-T serum concentration	Obesity	[146]
Alpha-T	Obese children (6–19 years old) showed lower serum concentration of alpha-T as demonstrated by the National Health and Examination Survey (NHANES III)	Obesity	[147]
Alpha-T and gamma-T3	Gamma-T3 (60 mg/kg of body weight/day), but not alpha-T, reduced the body fat mass of rats treated with glucocorticoid	Obesity	[148]
Alpha-T, alpha- and gamma-T3	Alpha- and gamma-T3, but not alpha-T, prevented differentiation of preadipocytes into adipocytes in 3T3-L1 cells	Obesity	[149]
Alpha-T	*Cis-trans* isomerization of phospholipid bilayers induced by thiyl radicals was inhibited by alpha-T	Cell membrane function	[150]

## References

[1] Shahidi F., Ambigaipalan P. (2015). Phenolics and polyphenolics in foods, beverages and spices: Antioxidant activity and health effects—A review. J. Funct. Foods.

[2] Zingg J.M. (2007). Modulation of signal transduction by vitamin E. Mol. Asp. Med..

[3] Shahidi F., Shukla V.K.S. (1996). Nontriacylglycerol constituents of fats, oils. Inf. Int. News Fats Oils Relat. Mater..

[4] Shin E.-C., Huang Y.-Z., Pegg R.B., Phillips R.D., Eitenmiller R.R. (2009). Commercial Runner peanut cultivars in the United States: Tocopherol composition. J. Agric. Food Chem..

[5] De Camargo A.C., Vieira T.M.F.S., Regitano-d’Arce M.A.B., de Alencar S.M., Calori-Domingues M.A., Canniatti-Brazaca S.G. (2012). Gamma radiation induced oxidation and tocopherols decrease in in-shell, peeled and blanched peanuts. Int. J. Mol. Sci..

[6] Rossi M., Alamprese C., Ratti S. (2007). Tocopherols and tocotrienols as free radical-scavengers in refined vegetable oils and their stability during deep-fat frying. Food Chem..

[7] Kornsteiner M., Wagner K.H., Elmadfa I. (2006). Tocopherols and total phenolics in 10 different nut types. Food Chem..

[8] Caporaso N., Savarese M., Paduano A., Guidone G., de Marco E., Sacchi R. (2015). Nutritional quality assessment of extra virgin olive oil from the Italian retail market: Do natural antioxidants satisfy EFSA health claims?. J. Food Compos. Anal..

[9] Karmowski J., Hintze V., Kschonsek J., Killenberg M., Böhm V. (2015). Antioxidant activities of tocopherols/tocotrienols and lipophilic antioxidant capacity of wheat, vegetable oils, milk and milk cream by using photochemiluminescence. Food Chem..

[10] Grilo E.C., Costa P.N., Gurgel C.S.S., Beserra A.F.L., Almeida F.N.S., Dimenstein R. (2014). Alpha-tocopherol and gamma-tocopherol concentration in vegetable oils. Food Sci. Technol. (Campinas).

[11] Schwartz H., Ollilainen V., Piironen V., Lampi A.-M. (2008). Tocopherol, tocotrienol and plant sterol contents of vegetable oils and industrial fats. J. Food Compos. Anal..

[12] Chun J., Lee J., Ye L., Exler J., Eitenmiller R.R. (2006). Tocopherol and tocotrienol contents of raw and processed fruits and vegetables in the United States diet. J. Food Compos. Anal..

[13] De Camargo A.C., Regitano-d’Arce M.A.B., Gallo C.R., Shahidi F. (2015). Gamma-irradiation induced changes in microbiological status, phenolic profile and antioxidant activity of peanut skin. J. Funct. Foods.

[14] Alshikh N., de Camargo A.C., Shahidi F. (2015). Phenolics of selected lentil cultivars: Antioxidant activities and inhibition of low-density lipoprotein and DNA damage. J. Funct. Foods.

[15] Ayoub M., de Camargo A.C., Shahidi F. (2016). Antioxidants and bioactivities of free, esterified and insoluble-bound phenolics from berry seed meals. Food Chem..

[16] De Camargo A.C., Regitano-d’Arce M.A.B., Biasoto A.C.T., Shahidi F. (2014). Low molecular weight phenolics of grape juice and winemaking byproducts: Antioxidant activities and inhibition of oxidation of human low-density lipoprotein cholesterol and DNA strand breakage. J. Agric. Food Chem..

[17] Pryor W.A. (2000). Vitamin E and heart disease: Basic science to clinical intervention trials. Free Radic. Biol. Med..

[18] Pryor W.A. (1991). The antioxidant nutrients and disease prevention—What do we know and what do we need to find out?. Am. J. Clin. Nutr..

[19] Burton G.W., Traber M.G., Acuff R.V., Walters D.N., Kayden H., Hughes L., Ingold K.U. (1998). Human plasma and tissue alpha-tocopherol concentrations in response to supplementation with deuterated natural and synthetic vitamin E. Am. J. Clin. Nutr..

[20] Carter C., Finley W., Fry J., Jackson D., Willis L. (2007). Palm oil markets and future supply. Eur. J. Lipid Sci. Technol..

[21] Syväoja E.-L., Pilronen V., Varo P., Koivistoinen P., Salminen K. (1986). Tocopherols and tocotrienols in finnish foods: Oils and fats. J. Am. Oil Chem. Soc..

[22] Velasco L., Perez-Vich B., Fernandez-Martinez J.M. (2004). Novel variation for the tocopherol profile in a sunflower created by mutagenesis and recombination. Plant Breed..

[23] Moreau R.A., Flores R.A., Hicks K.B. (2007). Composition of functional lipids in hulled and hulless barley in fractions obtained by scarification and in barley oil. Cereal Chem..

[24] Wang L., Newman R.K., Newman C.W., Jackson L.L., Hofer P.J. (1993). Tocotrienol and fatty acid composition of barley oil and their effects on lipid metabolism. Plant Foods Hum. Nutr..

[25] Desai I.D., Bhagavan H., Salkeld R., Dutra de Oliveira J.E. (1988). Vitamin E content of crude and refined vegetable oils in southern Brazil. J. Food Compos. Anal..

[26] Carpenter A.P. (1979). Determination of tocopherols in vegetable oils. J. Am. Oil Chem. Soc..

[27] Corsini M.S., Silva M.G., Jorge N. (2009). Loss in tocopherols and oxidative stability during the frying of frozen cassava chips. Grasas Aceites.

[28] Ng M.H., Choo Y.M., Ma A.N., Chuah C.H., Hashim M.A. (2004). Separation of vitamin E (tocopherol, tocotrienol, and tocomonoenol) in palm oil. Lipids.

[29] Goufo P., Trindade H. (2014). Rice antioxidants: Phenolic acids, flavonoids, anthocyanins, proanthocyanidins, tocopherols, tocotrienols, γ-oryzanol, and phytic acid. Food Sci. Nutr..

[30] Aued-Pimentel S., Takemoto E., Antoniassi R., Badolato E.S.G. (2006). Composition of tocopherols in sesame seed oil: An indicative of adulteration. Grasas Aceites.

[31] Warner K., Mounts T.L. (1990). Analysis of tocopherols and phytosterols in vegetable-oils by HPLC with evaporative light-scattering detection. J. Am. Oil Chem. Soc..

[32] Sofi F., Macchi C., Abbate R., Gensini G.F., Casini A. (2013). Mediterranean diet and health. Biofactors.

[33] De Camargo A.C., Canniatti-Brazaca S.G., Cook R.W. (2014). Peanuts as a source of protein, unsaturated fatty acids, tocopherol and polyphenols. Peanuts: Production, Nutritional Content and Health Implications.

[34] Da Silva A.C., Jorge N. (2014). Bioactive compounds of the lipid fractions of agro-industrial waste. Food Res. Int..

[35] Malacrida C.R., Jorge N. (2012). Yellow passion fruit seed oil (*Passiflora edulis* F. *flavicarpa*): Physical and chemical characteristics. Braz. Arch. Biol. Technol..

[36] Malacrida C.R., Kimura M., Jorge N. (2011). Characterization of a high oleic oil extracted from papaya (*Carica papaya* L.) seeds. Ciencia Tecnol. Aliment..

[37] Malacrida C.R., Kimura M., Jorge N. (2012). Phytochemicals and antioxidant activity of citrus seed oils. Food Sci. Technol. Res..

[38] Seppanen C.M., Song Q., Csallany A.S. (2010). The antioxidant functions of tocopherol and tocotrienol homologues in oils, fats, and food systems. J. Am. Oil Chem. Soc..

[39] Li Q., Wang J., Shahidi F. (2016). Chemical characteristics of cold-pressed blackberry, black raspberry, and blueberry seed oils and the role of the minor components in their oxidative stability. J. Agric. Food Chem..

[40] Coimbra M.C., Jorge N. (2012). Fatty acids and bioactive compounds of the pulps and kernels of Brazilian palm species, guariroba (*Syagrus oleraces*), jerivá (*Syagrus romanzoffiana*) and macaúba (*Acrocomia aculeata*). J. Sci. Food Agric..

[41] Dias L.S., Luzia D.M.M., Jorge N. (2013). Physicochemical and bioactive properties of *Hymenaea courbaril* L. pulp and seed lipid fraction. Ind. Crops Prod..

[42] Luzia D.M.M., Jorge N. (2013). Bioactive substance contents and antioxidant capacity of the lipid fraction of *Annona crassiflora* Mart. seeds. Ind. Crops Prod..

[43] Luzia D.M.M., Jorge N. (2011). Antioxidant activity, fatty acid profile and tocopherols of *Tamarindus indica* L. Seeds. Ciencia Tecnol. Aliment..

[44] Inada K.O.P., Oliveira A.A., Revoredo T.B., Martins A.B.N., Lacerda E.C.Q., Freire A.S., Braz B.F., Santelli R.E., Torres A.G., Perrone D. (2015). Screening of the chemical composition and occurring antioxidants in jabuticaba (*Myrciaria jaboticaba*) and jussara (*Euterpe edulis*) fruits and their fractions. J. Funct. Foods.

[45] Da Silva P.P.M., Casemiro R.C., Zillo R.R., de Camargo A.C., Prospero E.T.P., Spoto M.H.F. (2014). Sensory descriptive quantitative analysis of unpasteurized and pasteurized juçara pulp (*Euterpe edulis*) during long-term storage. Food Sci. Nutr..

[46] Morales P., Barros L., Dias M.I., Santos-Buelga C., Ferreira I.C.F.R., Asquieri E.R., Berrios J.D.J. (2016). Non-fermented and fermented jabuticaba (*Myrciaria cauliflora* Mart.) pomaces as valuable sources of functional ingredients. Food Chem..

[47] Darnet S., Serra J.L., da Cruz Rodrigues A.M., Meller da Silva L.H. (2011). A high-performance liquid chromatography method to measure tocopherols in assai pulp (*Euterpe oleracea*). Food Res. Int..

[48] Coimbra M.C., Jorge N. (2011). Characterization of the pulp and kernel oils from *Syagrus oleracea*, *Syagrus romanzoffiana*, and *Acrocomia aculeata*. J. Food Sci..

[49] Montoya C., Cochard B., Flori A., Cros D., Lopes R., Cuellar T., Espeout S., Syaputra I., Villeneuve P., Pina M. (2014). Genetic architecture of palm oil fatty acid composition in cultivated oil palm (*Elaeis guineensis* jacq.) compared to its wild relative E. oleifera (HBK) cortes. PLoS ONE.

[50] Warner K., Gupta M. (2005). Potato chip quality and frying oil stability of high oleic acid soybean oil. J. Food Sci..

[51] Merrill L.I., Pike O.A., Ogden L.V., Dunn M.L. (2008). Oxidative stability of conventional and high-oleic vegetable oils with added antioxidants. J. Am. Oil Chem. Soc..

[52] Shin E.-C., Craft B.D., Pegg R.B., Phillips R.D., Eitenmiller R.R. (2010). Chemometric approach to fatty acid profiles in Runner-type peanut cultivars by principal component analysis (PCA). Food Chem..

[53] Wilkin J.D., Ashton I.P., Fielding L.M., Tatham A.S. (2014). Storage stability of whole and nibbed, conventional and high oleic peanuts (*Arachis hypogeae* L.). Food Bioprocess Technol..

[54] Janila P., Pandey M.K., Shasidhar Y., Variath M.T., Sriswathi M., Khera P., Manohar S.S., Nagesh P., Vishwakarma M.K., Mishra G.P. (2016). Molecular breeding for introgression of fatty acid desaturase mutant alleles (*ahFAD2A* and *ahFAD2*B) enhances oil quality in high and low oil containing peanut genotypes. Plant Sci..

[55] Abidi S.L., List G.R., Rennick K.A. (1999). Effect of genetic modification on the distribution of minor constituents in canola oil. J. Am. Oil Chem. Soc..

[56] Dionisi F., Prodolliet J., Tagliaferri E. (1995). Assessment of olive oil adulteration by reversed-phase high-performance liquid chromatography amperometric detection of tocopherols and tocotrienols. J. Am. Oil Chem. Soc..

[57] Clemente T.E., Cahoon E.B. (2009). Soybean oil: Genetic approaches for modification of functionality and total content. Plant Physiol..

[58] Kim Y.H., Lee Y.Y., Choi M.S., Jeong K.H., Lee S.K., Seo M.J., Yun H.T., Lee C.K., Kim W.H., Lee S.C. (2011). Antioxidant activity and inhibition of lipid peroxidation in germinating seeds of transgenic soybean expressing OsHGGT. J. Agric. Food Chem..

[59] Tang Y., Li X.H., Chen P.X., Zhang B., Hernandez M., Zhang H., Marcone M.F., Liu R.H., Tsao R. (2015). Characterisation of fatty acid, carotenoid, tocopherol/tocotrienol compositions and antioxidant activities in seeds of three *Chenopodium quinoa* Wild genotypes. Food Chem..

[60] Shimada Y., Nakai S., Suenaga M., Sugihara A., Kitano M., Tominaga Y. (2000). Facile purification of tocopherols from soybean oil deodorizer distillate in high yield using lipase. J. Am. Oil Chem. Soc..

[61] Shahidi F., Zhong Y. (2010). Lipid oxidation and improving the oxidative stability. Chem. Soc. Rev..

[62] Pestana-Bauer V.R., Zambiazi R.C., Mendonca C.R.B., Beneito-Cambra M., Ramis-Ramos G. (2012). Gamma-oryzanol and tocopherol contents in residues of rice bran oil refining. Food Chem..

[63] Peterson D.M. (1995). Oat tocols—Concentration and stability in oat products and distribution within the kernel. Cereal Chem..

[64] Moreau R.A., Wayns K.E., Flores R.A., Hicks K.B. (2007). Tocopherols and tocotrienols in barley oil prepared from germ and other fractions from scarification and sieving of hulless barley. Cereal Chem..

[65] Kumar G.S., Krishna A.G.G. (2015). Studies on the nutraceuticals composition of wheat derived oils wheat bran oil and wheat germ oil. J. Food Sci. Technol..

[66] Zou L., Akoh C.C. (2015). Antioxidant activities of annatto and palm tocotrienol-rich fractions in fish oil and structured lipid-based infant formula emulsion. Food Chem..

[67] Shammugasamy B., Ramakrishnan Y., Manan F., Muhammad K. (2015). Rapid reversed-phase chromatographic method for determination of eight vitamin E isomers and γ-oryzanols in rice bran and rice bran oil. Food Anal. Methods.

[68] Chen B., McClements D.J., Decker E.A. (2011). Minor components in food oils: A critical review of their roles on lipid oxidation chemistry in bulk oils and emulsions. Crit. Rev. Food Sci. Nutr..

[69] Nagy K., Kerrihard A.L., Beggio M., Craft B.D., Pegg R.B. (2016). Modeling the impact of residual fat-soluble vitamin (FSV) contents on the oxidative stability of commercially refined vegetable oils. Food Res. Int..

[70] Huang S.W., Frankel E.N., German J.B. (1994). Antioxidant activity of α- and γ-tocopherols in bulk oils and in oil-in-water emulsions. J. Agric. Food Chem..

[71] Dolde D., Wang T. (2011). Oxidation of corn oils with spiked tocols. J. Am. Oil Chem. Soc..

[72] Wagner K.H., Wotruba F., Elmadfa I. (2001). Antioxidative potential of tocotrienols and tocopherols in coconut fat at different oxidation temperatures. Eur. J. Lipid Sci. Tech..

[73] Top A.G.M., Ong A.S.H., Kato A., Watanabe H., Kawada T. (1989). Antioxidant activities of palm vitamin E with special reference to tocotrienols. Elaeis.

[74] Melo P.S., Arrivetti L.D.O.R., de Alencar S.M., Skibsted L.H. (2016). Antioxidative and prooxidative effects in food lipids and synergism with α-tocopherol of açaí seed extracts and grape rachis extracts. Food Chem..

[75] Khan M.A., Shahidi F. (2000). Tocopherols and phospholipids enhance the oxidative stability of borage and evening primrose triacylglycerols. J. Food Lipids.

[76] Let M.B., Jacobsen C., Meyer A.S. (2007). Ascorbyl palmitate, gamma-tocopherol, and edta affect lipid oxidation in fish oil enriched salad dressing differently. J. Agric. Food Chem..

[77] Zou L., Akoh C.C. (2015). Oxidative stability of structured lipid-based infant formula emulsion: Effect of antioxidants. Food Chem..

[78] Zhang X., Shen Y., Prinyawiwatkul W., King J.M., Xu Z. (2013). Comparison of the activities of hydrophilic anthocyanins and lipophilic tocols in black rice bran against lipid oxidation. Food Chem..

[79] Xu Z.D., Harvey K.A., Pavlina T.M., Zaloga G.P., Siddiqui R.A. (2015). Tocopherol and tocotrienol homologs in parenteral lipid emulsions. Eur. J. Lipid Sci. Tech..

[80] Xu Z.D., Harvey K.A., Pavlina T.M., Zaloga G.P., Siddiqui R.A. (2016). Distribution of tocopherols and tocotrienols in guinea pig tissues following parenteral lipid emulsion infusion. J. Parenter. Enter. Nutr..

[81] Tian F., Decker E.A., Goddard J.M. (2013). Controlling lipid oxidation of food by active packaging technologies. Food Funct..

[82] Horn A.F., Nielsen N.S., Jacobsen C. (2009). Additions of caffeic acid, ascorbyl palmitate or γ-tocopherol to fish oil-enriched energy bars affect lipid oxidation differently. Food Chem..

[83] Kanatt S.R., Paul P., D’Souza S.F., Thomas P. (1998). Lipid peroxidation in chicken meat during chilled storage as affected by antioxidants combined with low-dose gamma irradiation. J. Food Sci..

[84] Barbosa-Pereira L., Cruz J.M., Sendón R., Rodríguez Bernaldo de Quirós A., Ares A., Castro-López M., Abad M.J., Maroto J., Paseiro-Losada P. (2013). Development of antioxidant active films containing tocopherols to extend the shelf life of fish. Food Control.

[85] Chen X., Lee D.S., Zhu X., Yam K.L. (2012). Release kinetics of tocopherol and quercetin from binary antioxidant controlled-release packaging films. J. Agric. Food Chem..

[86] Marcos B., Sárraga C., Castellari M., Kappen F., Schennink G., Arnau J. (2014). Development of biodegradable films with antioxidant properties based on polyesters containing α-tocopherol and olive leaf extract for food packaging applications. Food Packag. Shelf Life.

[87] Tricker A.R., Preussmann R. (1991). Carcinogenic *N*-nitrosamines in the diet: Occurrence, formation, mechanisms and carcinogenic potential. Mutat. Res./Genet. Toxicol..

[88] Yang H., Meng P., Xiong Y.L., Ma L., Wang C., Zhu Y. (2013). Oxidation in HiOx-packaged pork *Longissimus* muscle predisposes myofibrillar and sarcoplasmic proteins to *N*-nitrosamine formation in nitrite-curing solution. Meat Sci..

[89] Gray J.I., Skrypec D.J., Mandagere A.K., Booren A.M., Pearson A.M. (1984). Further factors influencing *N*-nitrosamine formation in bacon. IARC Sci. Publ..

[90] Kurechi T., Kikugawa K., Ozawa M. (1980). Effect of malondialdehyde on nitrosamine formation. Food Cosmet. Toxicol..

[91] Fiddler W., Pensabene J.W., Piotrowski E.G., Phillips J.G., Keating J., Mergens W.J., Newmark H.L. (1978). Inhibition of formation of volatile nitrosamines in fried bacon by the use of cure-solubilized α-tocopherol. J. Agric. Food Chem..

[92] Wang Y., Li F., Zhuang H., Chen X., Li L., Qiao W., Zhang J. (2015). Effects of plant polyphenols and α-tocopherol on lipid oxidation, residual nitrites, biogenic amines, and *N*-nitrosamines formation during ripening and storage of dry-cured bacon. LWT—Food Sci. Technol..

[93] Pourazrang H., Moazzami A.A., Bazzaz B.S.F. (2002). Inhibition of mutagenic N-nitroso compound formation in sausage samples by using L-ascorbic acid and α-tocopherol. Meat Sci..

[94] Pegg R.B., Shahidi F. (1997). Unraveling the chemical identity of meat pigments. Crit. Rev. Food Sci. Nutr..

[95] Djenane D., Sánchez-Escalante A., Beltrán J.A., Roncalés P. (2002). Ability of α-tocopherol, taurine and rosemary, in combination with vitamin C, to increase the oxidative stability of beef steaks packaged in modified atmosphere. Food Chem..

[96] Zhong Y., Lall S.P., Shahidi F. (2007). Effects of oxidized dietary oil and vitamin E supplementation on lipid profile and oxidation of muscle and liver of juvenile atlantic cod (*Gadus morhua*). J. Agric. Food Chem..

[97] Saberi A.H., Fang Y., McClements D.J. (2013). Fabrication of vitamin E-enriched nanoemulsions: Factors affecting particle size using spontaneous emulsification. J. Colloid Interface Sci..

[98] Hensley K., Benaksas E.J., Bolli R., Comp P., Grammas P., Hamdheydari L., Mou S., Pye Q.N., Stoddard M.F., Wallis G. (2004). New perspectives on vitamin E: γ-tocopherol and carboxyethylhydroxychroman metabolites in biology and medicine. Free Radic. Biol. Med..

[99] Reiter E., Jiang Q., Christen S. (2007). Anti-inflammatory properties of α- and γ-tocopherol. Mol. Asp. Med..

[100] Etminan M., Gill S.S., Samii A. (2005). Intake of vitamin E, vitamin C, and carotenoids and the risk of Parkinson’s disease: A meta-analysis. Lancet Neurol..

[101] Li F.J., Shen L., Ji H.F. (2012). Dietary intakes of vitamin E, vitamin C, and beta-carotene and risk of Alzheimer’s disease: A meta-analysis. J. Alzheimers Dis..

[102] De Camargo A.C., Regitano-d’Arce M.A.B., de Alencar S.M., Canniatti-Brazaca S.G., de Souza Vieira T.M.F., Shahidi F. (2016). Chemical changes and oxidative stability of peanuts as affected by the dry-blanching. J. Am. Oil Chem. Soc..

[103] Orem A., Yucesan F.B., Orem C., Akcan B., Kural B.V., Alasalvar C., Shahidi F. (2013). Hazelnut-enriched diet improves cardiovascular risk biomarkers beyond a lipid-lowering effect in hypercholesterolemic subjects. J. Clin. Lipidol..

[104] Di Stefano V., Pitonzo R., Bartolotta A., D’Oca M.C., Fuochi P. (2014). Effects of γ-irradiation on the α-tocopherol and fatty acids content of raw unpeeled almond kernels (*Prunus dulcis*). LWT—Food Sci. Technol..

[105] De Camargo A.C., Regitano-d’Arce M.A.B., de Souza T.M.F., de Alencar S.M., Gallo C.R., Spoto M.H.F., da Silva P.P.M., Canniatti-Brazaca S.G., Cook R.W. (2014). Gamma irradiation to improve microbiological safety of peanuts: Effects on peroxide value, HS-SPME-GC/MS hexanal content and sensory acceptance. Peanuts: Production, Nutritional Content and Health Implications.

[106] Sabliov C.M., Fronczek C., Astete C.E., Khachaturyan M., Khachatryan L., Leonardi C. (2009). Effects of temperature and UV light on degradation of α-tocopherol in free and dissolved form. J. Am. Oil Chem. Soc..

[107] Altunkaya A., Gökmen V., Skibsted L.H. (2016). pH dependent antioxidant activity of lettuce (*L. sativa*) and synergism with added phenolic antioxidants. Food Chem..

[108] Thoo Y.Y., Abas F., Lai O.-M., Ho C.W., Yin J., Hedegaard R.V., Skibsted L.H., Tan C.P. (2013). Antioxidant synergism between ethanolic centella asiatica extracts and α-tocopherol in model systems. Food Chem..

[109] Da Silva A.C., Jorge N. (2014). Influence of *Lentinus edodes* and *Agaricus blazei* extracts on the prevention of oxidation and retention of tocopherols in soybean oil in an accelerated storage test. J. Food Sci. Technol..

[110] Chandrasekara A., Shahidi F. (2011). Determination of antioxidant activity in free and hydrolyzed fractions of millet grains and characterization of their phenolic profiles by HPLC-DAD-ESI-MS^n^. J. Funct. Foods.

[111] De Camargo A.C., Regitano-d’Arce M.A.B., Biasoto A.C.T., Shahidi F. (2016). Enzyme-assisted extraction of phenolics from winemaking by-products: Antioxidant potential and inhibition of alpha-glucosidase and lipase activities. Food Chem..

[112] Saito Y., Nishio K., Akazawa Y.O., Yamanaka K., Miyama A., Yoshida Y., Noguchi N., Niki E. (2010). Cytoprotective effects of vitamin E homologues against glutamate-induced cell death in immature primary cortical neuron cultures: Tocopherols and tocotrienols exert similar effects by antioxidant function. Free Radic. Biol. Med..

[113] Terasawa Y., Ladha Z., Leonard S.W., Morrow J.D., Newland D., Sanan D., Packer L., Traber M.G., Farese R.V. (2000). Increased atherosclerosis in hyperlipidemic mice deficient in α-tocopherol transfer protein and vitamin E. Proc. Natl. Acad. Sci. USA.

[114] Hosomi A., Arita M., Sato Y., Kiyose C., Ueda T., Igarashi O., Arai H., Inoue K. (1997). Affinity for α-tocopherol transfer protein as a determinant of the biological activities of vitamin E analogs. FEBS Lett..

[115] Mah E., Sapper T.N., Chitchumroonchokchai C., Failla M.L., Schill K.E., Clinton S.K., Bobe G., Traber M.G., Bruno R.S. (2015). Alpha-tocopherol bioavailability is lower in adults with metabolic syndrome regardless of dairy fat co-ingestion: A randomized, double-blind, crossover trial. Am. J. Clin. Nutr..

[116] Drotleff A.M., Bohnsack C., Schneider I., Hahn A., Ternes W. (2014). Human oral bioavailability and pharmacokinetics of tocotrienols from tocotrienol-rich (tocopherol-low) barley oil and palm oil formulations. J. Funct. Foods.

[117] Madhujith T., Shahidi F. (2007). Antioxidative and antiproliferative properties of selected barley (*Hordeum vulgarae* L.) cultivars and their potential for inhibition of low-density lipoprotein (LDL) cholesterol oxidation. J. Agric. Food Chem..

[118] Salah N., Miller N.J., Paganga G., Tijburg L., Bolwell G.P., Riceevans C. (1995). Polyphenolic flavanols as scavengers of aqueous phase radicals and as chain-breaking antioxidants. Arch. Biochem. Biophys..

[119] Zhong Y., Ma C.-M., Shahidi F. (2012). Antioxidant and antiviral activities of lipophilic epigallocatechin gallate (EGCG) derivatives. J. Funct. Foods.

[120] Krumova K., Friedland S., Cosa G. (2012). How lipid unsaturation, peroxyl radical partitioning, and chromanol lipophilic tail affect the antioxidant activity of α-tocopherol: Direct visualization via high-throughput fluorescence studies conducted with fluorogenic α-tocopherol analogues. J. Am. Oil Chem. Soc..

[121] Rice-Evans C., Miller N., Paganga G. (1997). Antioxidant properties of phenolic compounds. Trends Plant Sci..

[122] Jialal I., Fuller C.J., Huet B.A. (1995). The effect of α-tocopherol supplementation on LDL oxidation: A dose-response study. Arterioscler. Thromb. Vasc. Biol..

[123] Roberts Ii L.J., Oates J.A., Linton M.F., Fazio S., Meador B.P., Gross M.D., Shyr Y., Morrow J.D. (2007). The relationship between dose of vitamin E and suppression of oxidative stress in humans. Free Radic. Biol. Med..

[124] Loffredo L., Perri L., di Castelnuovo A., Iacoviello L., de Gaetano G., Violi F. (2015). Supplementation with vitamin E alone is associated with reduced myocardial infarction: A meta-analysis. Nutr. Metab. Cardiovasc. Dis..

[125] World Health Organization Cardiovascular Disease. http://www.who.int/cardiovascular_diseases/en/.

[126] Christen S., Woodall A.A., Shigenaga M.K., SouthwellKeely P.T., Duncan M.W., Ames B.N. (1997). Gamma-tocopherol traps mutagenic electrophiles such as NOx and complements alpha-tocopherol: Physiological implications. Proc. Natl. Acad. Sci. USA.

[127] Abid-Essefi S., Baudrimont I., Hassen W., Ouanes Z., Mobio T.A., Anane R., Creppy E.E., Bacha H. (2003). DNA fragmentation, apoptosis and cell cycle arrest induced by zearalenone in cultured DOK, Vero and Caco-2 cells: Prevention by vitamin E. Toxicology.

[128] Campbell S.E., Stone W.L., Lee S., Whaley S., Yang H.S., Qui M., Goforth P., Sherman D., McHaffie D., Krishnan K. (2006). Comparative effects of RRR-alpha- and RRR-gamma-tocopherol on proliferation and apoptosis in human colon cancer cell lines. BMC Cancer.

[129] Nam J.-P., Lee K.-J., Choi J.-W., Yun C.-O., Nah J.-W. (2015). Targeting delivery of tocopherol and doxorubicin grafted-chitosan polymeric micelles for cancer therapy: In vitro and in vivo evaluation. Colloids Surf. B.

[130] Drotleff A.M., Büsing A., Willenberg I., Empl M.T., Steinberg P., Ternes W. (2015). HPLC separation of vitamin E and its oxidation products and effects of oxidized tocotrienols on the viability of MCF-7 breast cancer cells in vitro. J. Agric. Food Chem..

[131] Pierre F.H.F., Martin O.C.B., Santarelli R.L., Tache S., Naud N., Gueraud F., Audebert M., Dupuy J., Meunier N., Attaix D. (2013). Calcium and alpha-tocopherol suppress cured-meat promotion of chemically induced colon carcinogenesis in rats and reduce associated biomarkers in human volunteers. Am. J. Clin. Nutr..

[132] Kong P.F., Cai Q.Q., Geng Q.R., Wang J., Lan Y.D., Zhan Y.Q., Xu D.Z. (2014). Vitamin intake reduce the risk of gastric cancer: Meta-analysis and systematic review of randomized and observational studies. PLoS ONE.

[133] Virtamo J., Taylor P.R., Kontto J., Männistö S., Utriainen M., Weinstein S.J., Huttunen J., Albanes D. (2014). Effects of α-tocopherol and β-carotene supplementation on cancer incidence and mortality: 18-year postintervention follow-up of the alpha-tocopherol, beta-carotene cancer prevention study. Int. J. Cancer.

[134] Menkes M.S., Comstock G.W., Vuilleumier J.P., Helsing K.J., Rider A.A., Brookmeyer R. (1986). Serum beta-carotene, vitamins A and E, selenium, and the risk of lung cancer. N. Engl. J. Med..

[135] Yu W., Simmons-Menchaca M., Gapor A., Sanders B.G., Kline K. (1999). Induction of apoptosis in human breast cancer cells by tocopherols and tocotrienols. Nutr. Cancer.

[136] Wada S., Satomi Y., Murakoshi M., Noguchi N., Yoshikawa T., Nishino H. (2005). Tumor suppressive effects of tocotrienol in vivo and in vitro. Cancer Lett..

[137] Tam K.-W., Ho C.-T., Lee W.-J., Tu S.-H., Huang C.-S., Chen C.-S., Lee C.-H., Wu C.-H., Ho Y.-S. (2013). Alteration of α-tocopherol-associated protein (TAP) expression in human breast epithelial cells during breast cancer development. Food Chem..

[138] Hu F., Wu Z., Li G., Teng C., Liu Y., Wang F., Zhao Y., Pang D. (2015). The plasma level of retinol, vitamins A, C and α-tocopherol could reduce breast cancer risk? A meta-analysis and meta-regression. J. Cancer Res. Clin. Oncol..

[139] Kalender Y., Yel M., Kalender S. (2005). Doxorubicin hepatotoxicity and hepatic free radical metabolism in rats: The effects of vitamin E and catechin. Toxicology.

[140] Guthrie N., Gapor A., Chambers A.F., Carroll K.K. (1997). Inhibition of proliferation of estrogen receptor-negative MDA-MB-435 and -positive MCF-7 human breast cancer cells by palm oil tocotrienols and tamoxifen, alone and in combination. J. Nutr..

[141] Pathak R., Bachri A., Ghosh S.P., Koturbash I., Boerma M., Binz R.K., Sawyer J.R., Hauer-Jensen M. (2016). The vitamin E analog gamma-tocotrienol (Gt3) suppresses radiation-induced cytogenetic damage. Pharm. Res..

[142] Fang F., Kang Z., Wong C. (2010). Vitamin E tocotrienols improve insulin sensitivity through activating peroxisome proliferator-activated receptors. Mol. Nutr. Food Res..

[143] Bharti S.K., Kumar A., Sharma N.K., Prakash O., Jaiswal S.K., Krishnan S., Gupta A.K., Kumar A. (2013). Tocopherol from seeds of *Cucurbita pepo* against diabetes: Validation by in vivo experiments supported by computational docking. J. Formos. Med. Assoc..

[144] Knekt P., Reunanen A., Marniemi J., Leino A., Aromaa A. (1999). Low vitamin E status is a potential risk factor for insulin-dependent diabetes mellitus. J. Intern. Med..

[145] Sato K., Gosho M., Yamamoto T., Kobayashi Y., Ishii N., Ohashi T., Nakade Y., Ito K., Fukuzawa Y., Yoneda M. (2015). Vitamin E has a beneficial effect on nonalcoholic fatty liver disease: A meta-analysis of randomized controlled trials. Nutrition.

[146] Botella-Carretero J.I., Balsa J.A., Vazquez C., Peromingo R., Diaz-Enriquez M., Escobar-Morreale H.F. (2010). Retinol and alpha-tocopherol in morbid obesity and nonalcoholic fatty liver disease. Obes. Surg..

[147] Strauss R.S. (1999). Comparison of serum concentrations of α-tocopherol and β-carotene in a cross-sectional sample of obese and nonobese children (NHANES III). J. Pediatr..

[148] Ima-Nirwana S., Suhaniza S. (2004). Effects of tocopherols and tocotrienols on body composition and bone calcium content in adrenalectomized rats replaced with dexamethasone. J. Med. Food..

[149] Uto-Kondo H., Ohmori R., Kiyose C., Kishimoto Y., Saito H., Igarashi O., Kondo K. (2009). Tocotrienol suppresses adipocyte differentiation and Akt phosphorylation in 3t3-L1 preadipocytes. J. Nutr..

[150] Chatgilialoglu C., Zambonin L., Altieri A., Ferreri C., Mulazzani Q.G., Landi L. (2002). Geometrical isomerism of monounsaturated fatty acids: Thiyl radical catalysis and influence of antioxidant vitamins. Free Radic. Biol. Med..

[151] Mozaffarian D., Katan M.B., Ascherio A., Stampfer M.J., Willett W.C. (2006). *Trans* fatty acids and cardiovascular disease. N. Engl. J. Med..

[152] Hung W.-L., Sun Hwang L., Shahidi F., Pan M.-H., Wang Y., Ho C.-T. (2016). Endogenous formation of *trans* fatty acids: Health implications and potential dietary intervention. J. Funct. Foods.

[153] Zhao M.L., Tang L., Zhu X.M., Hu J.N., Li H.Y., Luo L.P., Lei L., Deng Z.Y. (2013). Enzymatic production of zero-trans plastic fat rich in α-linolenic acid and medium-chain fatty acids from highly hydrogenated soybean oil, cinnamomum camphora seed oil, and perilla oil by lipozyme TL IM. J. Agric. Food Chem..

[154] Kemény Z., Recseg K., Hénon G., Kovári K., Zwobada F. (2001). Deodorization of vegetable oils: Prediction of trans polyunsaturated fatty acid content. J. Am. Oil Chem. Soc..

[155] Milstien S., Katusic Z. (1999). Oxidation of tetrahydrobiopterin by peroxynitrite: Implications for vascular endothelial function. Biochem. Biophys. Res. Commun..

[156] McCarty M.F. (2007). Gamma-tocopherol may promote effective no synthase function by protecting tetrahydrobiopterin from peroxynitrite. Med. Hypotheses.

[157] International Agency for Research on Cancer (IARC) World Cancer Factsheet. http://publications.cancerresearchuk.org/downloads/Product/CS_REPORT_WORLD.pdf.

[158] Calori-Domingues M.A., Teles D., Ponce G.H., de Camargo A.C., Dias C.T.S., da Gloria E.M., Aguila L.S.H., de Godoy I.J., Cook R.W. (2014). A non-toxigenic strain of *Aspergillus flavus* as a biological control against aflatoxin production in peanuts. Peanuts: Production, Nutritional Content and Health Implications.

[159] Santili A.B.N., de Camargo A.C., Nunes R.S.R., Gloria E.M.d., Machado P.F., Cassoli L.D., Dias C.T.S., Calori-Domingues M.A. (2015). Aflatoxin M1 in raw milk from different regions of São Paulo state—Brazil. Food Addit. Contam. B.

[160] Calori-Domingues M.A., Bernardi C.M.G., Nardin M.S., de Souza G.V., dos Santos F.G.R., Stein M.A., da Gloria E.M., Dias C.T.S., de Camargo A.C. (2016). Co-occurrence and distribution of deoxynivalenol, nivalenol and zearalenone in wheat from Brazil. Food Addit. Contam. B.

[161] Miranda D.D.C., Arçari D.P., Ladeira M.S.P., Calori-Domingues M.A., Romero A.C., Salvadori D.M.F., Gloria E.M., Pedrazzoli J., Ribeiro M.L. (2007). Analysis of DNA damage induced by aflatoxin B1 in Dunkin-Hartley guinea pigs. Mycopathologia.

[162] Huang H., He Y., Cui X.-X., Goodin S., Wang H., Du Z.Y., Li D., Zhang K., Tony Kong A.-N., DiPaola R.S. (2014). Potent inhibitory effect of δ-tocopherol on prostate cancer cells cultured in vitro and grown as xenograft tumors in vivo. J. Agric. Food Chem..

[163] Corpet D.E. (2011). Red meat and colon cancer: Should we become vegetarians, or can we make meat safer?. Meat Sci..

[164] Cheung-Ong K., Giaever G., Nislow C. (2013). DNA-damaging agents in cancer chemotherapy: Serendipity and chemical biology. Chem. Biol..

[165] Šimůnek T., Štěrba M., Popelová O., Adamcová M., Hrdina R., Geršl V. (2009). Anthracycline-induced cardiotoxicity: Overview of studies examining the roles of oxidative stress and free cellular iron. Pharmacol. Rep..

[166] Ambigaipalan P., de Camargo A.C., Shahidi F. (2016). Phenolic compounds of pomegranate by-products (outer skin, mesocarp, divider membrane) and their antioxidant activities. J. Agric. Food Chem..

[167] Mayer P.J., Lange C.S., Bradley M.O., Nichols W.W. (1989). Age-dependent decline in rejoining of X-ray-induced DNA double-strand breaks in normal human lymphocytes. Mutat. Res..

[168] Singh N.P., Danner D.B., Tice R.R., Brant L., Schneider E.L. (1990). DNA damage and repair with age in individual human lymphocytes. Mutat. Res..

[169] Green M.H.L., Lowe J.E., Waugh A.P.W., Aldridge K.E., Cole J., Arlett C.F. (1994). Effect of diet and vitamin C on DNA strand breakage in freshly-isolated human white blood cells. Mutat. Res..

[170] Zhang B., Deng Z., Ramdath D.D., Tang Y., Chen P.X., Liu R., Liu Q., Tsao R. (2015). Phenolic profiles of 20 canadian lentil cultivars and their contribution to antioxidant activity and inhibitory effects on α-glucosidase and pancreatic lipase. Food Chem..

[171] Sun S., Kadouh H.C., Zhu W., Zhou K. (2016). Bioactivity-guided isolation and purification of α-glucosidase inhibitor, 6-*O*-*D*-glycosides, from tinta Cão grape pomace. J. Funct. Foods.

[172] Zhang Y.F., Jiang W.J., Xie Z.T., Wu W.L., Zhang D.F. (2015). Vitamin E and risk of age-related cataract: A meta-analysis. Public Health Nutr..

[173] Lu Y., Demleitner M.F., Song L., Rychlik M., Huang D. (2016). Oligomeric proanthocyanidins are the active compounds in *Abelmoschus* esculentus *Moench* for its α-amylase and α-glucosidase inhibition activity. J. Funct. Foods.

[174] Mayer-Davis E.J., Costacou T., King I., Zaccaro D.J., Bell R.A. (2002). Plasma and dietary vitamin E in relation to incidence of type 2 diabetes—The insulin resistance and atherosclerosis study (IRAS). Diabetes Care.

[175] Rösen P., Nawroth P.P., King G., Möller W., Tritschler H.J., Packer L. (2001). The role of oxidative stress in the onset and progression of diabetes and its complications: Asummary of a congress series sponsored by UNESCO-MCBN, the American Diabetes Association and the German Diabetes Society. Diabetes Metab. Res. Rev..

[176] World Health Organization Obesity. http://www.who.int/topics/obesity/en/.

[177] Wang Y.F., Monteiro C., Popkin B.M. (2002). Trends of obesity and underweight in older children and adolescents in the United States, Brazil, China, and Russia. Am. J. Clin. Nutr..

[178] Schmidt M.I., Duncan B.B., Silva G.A.E., Menezes A.M., Monteiro C.A., Barreto S.M., Chor D., Menezes P.R. (2011). Chronic non-communicable diseases in Brazil: Burden and current challenges. Lancet.

[179] Mendez M.A., Monteiro C.A., Popkin B.M. (2005). Overweight exceeds underweight among women in most developing countries. Am. J. Clin. Nutr..

[180] Monteiro C.A., Conde W.L., Popkin B.M. (2001). Independent effects of income and education on the risk of obesity in the Brazilian adult population. J. Nutr..

[181] Adams L.A., Lindor K.D. (2007). Nonalcoholic fatty liver disease. Ann. Epidemiol..

[182] Zhao L., Fang X., Marshall M.R., Chung S. (2016). Regulation of obesity and metabolic complications by gamma and delta tocotrienols. Molecules.

[183] Kawai Y., Shimomitsu T., Takanami Y., Murase N., Katsumura T., Maruyama C. (2000). Vitamin E level changes in serum and red blood cells due to acute exhaustive exercise in collegiate women. J. Nutr. Sci. Vitaminol..

[184] Balazy M., Chemtob S. (2008). Trans-arachidonic acids: New mediators of nitro-oxidative stress. Pharmacol. Ther..

[185] Zambonin L., Ferreri C., Cabrini L., Prata C., Chatgilialoglu C., Landi L. (2006). Occurrence of trans fatty acids in rats fed a trans-free diet: A free radical-mediated formation?. Free Radic. Biol. Med..

[186] Zambonin L., Prata C., Cabrini L., Maraldi T., Fiorentini D., Sega F.V.D., Hakim G., Landi L. (2008). Effect of radical stress and ageing on the occurrence of trans fatty acids in rats fed a trans-free diet. Free Radic. Biol. Med..

[187] Ferreri C., Costantino C., Chatgilialoglu C., Landi L., Mulazzani Q.G. (1999). The thiyl radical-mediated isomerization of cis-monounsaturated fatty acid residues in phospholipids: A novel path of membrane damage?. Chem. Commun..

[188] Ferreri C., Costantino C., Perrotta L., Landi L., Mulazzani Q.G., Chatgilialoglu C. (2001). Cis-trans isomerization of polyunsaturated fatty acid residues in phospholipids catalyzed by thiyl radicals. J. Am. Oil Chem. Soc..

[189] Konings A.W.T., Damen J., Trieling W.B. (1979). Protection of liposomal lipids against radiation-induced oxidative damage. Int. J. Radiat. Biol..

